# Faecal microbiome, gastrointestinal integrity, inflammation and thermoregulation in recent exertional heat illness patients and matched controls

**DOI:** 10.1113/EP092849

**Published:** 2025-06-23

**Authors:** Alex A. M. Gould, Neil P. Walsh, Michael J. Tipton, Michael J. Zurawlew, Omar Tayari, Carol House, Simon K. Delves, Samuel C. Robson, Janis J. Shute, Joy E. M. Watts, Andrew J. Roberts, Alex J. Rawcliffe, Megan R. Robinson, Jo Corbett

**Affiliations:** ^1^ Extreme Environments Laboratory, School of Psychology, Sport, and Health Sciences, Faculty of Science and Health University of Portsmouth Portsmouth UK; ^2^ School of Sport and Exercise Sciences Liverpool John Moores University Liverpool UK; ^3^ Environmental Medicine and Science Division Institute of Naval Medicine Alverstoke UK; ^4^ Institute of Life Sciences and Healthcare & Centre for Enzyme Innovation University of Portsmouth Portsmouth UK; ^5^ School of Medicine, Pharmacy and Biomedical Sciences, Faculty of Science and Health University of Portsmouth Portsmouth UK; ^6^ School of the Environment and Life Sciences, Faculty of Science and Health University of Portsmouth Portsmouth UK; ^7^ Army Recruit Health and Performance Research, Medical Branch HQ Army Recruiting and Initial Training Command, Ministry of Defence Upavon UK

**Keywords:** exercise induced gastrointestinal syndrome, gastrointestinal paradigm, heat stroke, intestinal epithelial hyperpermeability, military

## Abstract

The gastrointestinal (GI) microbiota and GI barrier integrity are hypothesised to contribute to exertional heat illness (EHI) aetiology. We compared the faecal microbiome, GI barrier integrity, inflammation and thermoregulation of 29 recent (∼4 months) EHI patients (a group with elevated EHI risk) and 29 control individuals without prior EHI history, matched for variables influencing thermoregulation and GI microbiota. Participants completed an exercise heat tolerance assessment (HTA), with faecal microbiome assessed by 16S rRNA gene amplicon sequencing of stool samples and blood biomarkers of GI barrier integrity and inflammation measured pre‐ and post‐HTA. With the exception of the Simpson index (patient = 0.97 ± 0.01 vs. control = 0.98 ± 0.00, *P *= 0.030), there were no between‐groups differences in faecal microbiome composition (α‐diversity, β‐diversity, relative abundance, differential abundance), GI barrier integrity, inflammation or terminal thermoregulatory indices. Individuals were subsequently classified as heat tolerant (*n *= 46) or intolerant (*n *= 12) on the basis of the HTA. Heat intolerant individuals demonstrated lower sudomotor response (intolerant = 0.53 (0.17) vs. tolerant = 0.62 (0.20) L m^−2^ h^−1^, *P *= 0.011) despite greater thermoregulatory strain (e.g., terminal *T*_rec_: intolerant = 39.20 ± 0.31 vs. tolerant = 38.80 ± 0.31°C, *P *< 0.001), lower Firmicutes:Bacteroidota ratio (intolerant = 3.7 (0.6) vs. tolerant = 4.5 (2.0), *P *= 0.019) and higher plasma [sCD14] (*P *= 0.014), but other aspects of faecal microbiome, GI integrity or inflammation did not differ from heat tolerant individuals. In conclusion, the faecal microbiome composition and the GI barrier integrity and inflammatory responses to exercise heat‐stress showed limited differences between recent EHI patients and matched controls, or between individuals classified as heat intolerant or heat tolerant and are unlikely to explain elevated EHI risk in recent EHI patients, or heat intolerance.

## Abstract (highlights)

- **What is the central question of this study?**
  Do the gastrointestinal (GI) microbiota and GI barrier integrity contribute to exertional heat illness (EHI) aetiology?
- **What is the main finding and its importance?**
  Some individuals in both the EHI patient and control groups demonstrated heat intolerance; these individuals also had reduced sweating rate and elevated plasma [sCD14], but limited differences in the faecal microbiome composition. The composition of the faecal microbiome appears unlikely to explain elevated EHI risk in recent EHI patients, or heat intolerance and its assessment does not appear warranted within a return to duty evaluation.

## 1 INTRODUCTION

Exertional heat illnesses (EHIs) are medical conditions commonly described as being linked to strenuous exercise and high body temperature (Ministry of Defence, 2020; Roberts et al., 2021) and classified across a spectrum of increasing severity ranging from exertional heat exhaustion to exertional heat injury and exertional heat stroke (EHS) (Laitano et al., 2019). Severe EHIs may be fatal (Porter, 2000; Rav‐Acha et al., 2004), but are also associated with an increased risk of adverse sequalae including cardiovascular disease (Wang et al., 2019), chronic kidney disease (Tseng et al., 2020), neurological conditions (Lawton et al., 2019) and earlier all‐cause mortality (Wallace et al., 2007). EHIs remain an ongoing concern for those undertaking physically demanding activities under thermally stressful conditions (Alele et al., 2020; Bonauto et al., 2007; Gosling et al., 2008; Kerr et al., 2013).

Understanding of the factors precipitating an EHI episode is incomplete. Although putative EHI risk factors have been identified, the quality of underpinning evidence is generally limited (Westwood et al., 2021), with commonly identified risk factors (e.g., lack of heat acclimatisation, dehydration, inter‐current illness, low fitness) absent in approximately half of 361 EHI cases reported within UK Defence between 2007 and 2014 (Stacey et al., 2015). Moreover, once an individual has suffered an EHI episode they are at an elevated risk of reoccurrence (Nelson et al., 2018a; Nelson et al., 2018b; Stearns et al., 2020). Together, this suggests that alternative risk factors, which may present chronically, are important within EHI aetiology and could contribute to an ongoing elevated susceptibility risk (Hosokawa et al., 2019).

Recent work has hypothesised a potential role for the gastrointestinal (GI) system in the aetiology of EHI (Armstrong et al., 2018; Costa et al., 2017; Garcia et al., 2022; Lim, 2018; Ogden, Child et al., 2020). According to these GI models of EHI, sustained exertional heat strain causes disturbances to GI integrity as a result of splanchnic hypoperfusion (Kenney & Ho, 1995; Rehrer *et al.*, 2001; van Wijck et al., 2011) and neuroendocrine‐instigated intestinal epithelial injury and hyperpermeability (Costa *et al.*, 2022). This can increase microbial translocation from the GI tract into the circulating blood (Henningsen et al., 2024) and trigger downstream responses that induce acute inflammation (Iwaniec et al., 2021) and may lead to the systemic inflammatory response which precipitates the tissue and multi‐organ damage characteristic of the most severe forms of EHI (Carvalho et al., 2016; Li et al., 2022; Ubaldo et al., 2020).

The GI tract extends from the stomach to the colon, forming a 250–400 m^2^ interface between the GI lumen and circulating blood (Thursby & Juge, 2017) and containing a collection of microorganisms (bacteria, archaea, eukaryotes, viruses) which constitute the GI microbiota, with their associated genomes termed the GI microbiome (Ursell et al., 2012). The GI microbiome composition varies considerably between individuals, but demonstrates good stability over time and has trait‐like characteristics (Lozupone et al., 2012; Zoetendal et al., 1998). The gut microbiota exert a wide range of immunological, metabolic, structural and neurological effects on host physiology (Adak & Khan, 2019). Whilst the precise characteristics of a ‘healthy’ microbiome are still to be elucidated (Van Hul et al., 2024) the overall diversity of the GI microbiome may be an important marker of ‘health’ (Le Chatelier et al., 2013). Insights into GI microbiome composition are commonly obtained from measurement of the faecal microbiome, with higher faecal microbiome diversity reported in athletes compared to healthy controls (Clarke et al., 2014; Kulecka et al., 2020; Mörkl et al., 2017) and lower diversity reported in individuals with inflammatory bowel disease (Manichanh et al., 2006), type 1 and type 2 diabetes (De Goffau et al., 2013; Lambeth et al., 2015) and obesity (Turnbaugh et al., 2009; Verdam et al., 2013). Likewise, microbial diversity, as well as the relative abundance of certain bacterial taxa, has been associated with GI epithelial barrier integrity and systemic inflammation (Baumgart & Carding, 2007; Bennett et al., 2020; Ghosh et al., 2021; Li et al., 2021; Scheithauer et al., 2020) as well as temperature regulation (Bongers et al., 2023; Conn et al., 1991; Kluger et al., 1990; Li et al., 2021) – factors also implicated in the GI model of EHI (Ogden, Child et al., 2020). Consequently, it has been suggested that the GI microbiome may play a role in EHI aetiology (Armstrong et al., 2012, 2018; Roberts et al., 2021), but relevant empirical human studies are lacking.

To advance our understanding of EHI, it has been recommended that research should examine cohorts who have suffered a recent EHI episode, including individuals with ongoing impaired heat tolerance (Hosokawa et al., 2019). Within the UK, service personnel who have sustained a significant EHI are medically downgraded and may be required to undertake a Heat Tolerance Assessment (HTA) (House et al., 2021). The HTA assesses the thermoregulatory response to exercise heat‐stress and aids the return to exercise, training and occupational duty recommendations for individuals who have suffered EHI. Although most individuals achieve thermal balance, a proportion of individuals demonstrate heat intolerance and are referred for further investigation (House et al., 2021). This assessment, therefore, provides the unique opportunity to investigate the GI model in a cohort likely to have elevated EHI susceptibility and variation in heat tolerance.

Accordingly, our primary aim was to compare the faecal microbiome composition and indices of GI barrier integrity, inflammation and thermoregulation during exercise heat stress between individuals with a recent EHI and matched controls with no prior EHI history. The secondary aim was to compare the faecal microbiome composition and indices of GI barrier integrity, inflammation and thermoregulation during exercise heat stress between individuals inferred as heat tolerant and individuals inferred as heat intolerant. We hypothesised that: (i) the composition of the faecal microbiome would differ between patients and matched controls (e.g., lower diversity in patients); (ii) disturbances to GI barrier integrity, microbial translocation and inflammation would be greater during a HTA in recent EHI patients compared to matched controls; (iii) the composition of the faecal microbiome would differ between individuals inferred as heat tolerant compared to heat intolerant individuals (e.g., lower diversity in heat intolerant); and (iv) disturbances to GI barrier integrity, microbial translocation, and inflammation would be greater during an HTA in individuals inferred as heat intolerant compared to heat tolerant individuals.

## 2 METHODS

### 2.1 Ethical approval

The study protocol was approved by the Ministry of Defence Research Ethics Committee (Protocol number 2093/MODREC/21) and registered (clinicaltrials.gov: NCT05303142). The study was conducted in accordance with the *Declaration of Helsinki*. All participants received a detailed briefing on the purpose, nature and potential risks involved with the protocol prior to participating. Written informed consent was obtained from all participants; for logistical reasons some participants initially provided verbal informed consent to participate (recorded), subsequent to providing their written informed consent upon attendance at the Institute of Naval Medicine (INM).

### 2.2 Study design

The study employed a cross‐sectional design to compare the faecal microbiome and GI barrier integrity, inflammation and thermoregulation between a group of recent EHI patients (Patients) and a group of matched controls (Controls). Sample size estimation for microbiome research is challenging and remains rare (Debelius et al., 2016; Knight et al., 2018). Our sample size calculation was informed by Clarke et al. (2014) and indicated that a sample size of 58 would be sufficient to detect a medium effect size (*d *= 0.75) between groups for α‐diversity of the gut microbiome with an α of 0.05, an allocation ratio of 1 (i.e., 29 per group) and an observed power (1 − β) of 0.8 (G*power 3.1.7). Secondary outcome measures were also considered, with a minimum sample size of 18 per group sufficient for detection of previously reported between‐group differences in Δ intestinal fatty acid binding protein (I‐FABP), the most commonly employed marker of GI epithelial damage (Ogden, Fallowfield, Child, Davison, Fleming, Delves et al., 2020).

### 2.3 Participants

Recent EHI patients were recruited from individuals attending the INM Heat Illness Clinic (HIC), between February 2022 and March 2023. Male and female patients, aged between 18 and 45 years with a normal resting electrocardiogram (ECG) and diagnosis of recent category B (heat illness requiring admission to hospital with either central nervous system disturbance (e.g., seizure, Glasgow Coma Scale < 8 for 15 min or longer) and/or biochemical evidence of organ damage or rhabdomyolysis), category C (severe heat illness requiring admission to intensive care), or category D (more than one lifetime episode of heat illness) heat illness, were eligible for participation. EHI patients were not eligible for participation if they were: classified as category A heat illness (mild heat illness not requiring admission to hospital; no evidence of biochemical abnormality, no concurrent predisposing illness); recently diagnosed as or suspected to be hyponatraemic or suffering exertional rhabdomyolysis; or referred following exertional collapse without evidence of overheating.

Control participants were military personnel aged between 18 and 45 years with a normal resting electrocardiogram and no previous history of EHI; female control participants were not recruited due to absence of female representation in the EHI patient cohort. Military control participants were recruited rather than civilian controls to ensure familiarity with the load carriage undertaken within the HTA and to control for EHI ‘exposure risk’. The EHI patient and control cohorts were matched for factors known to influence thermoregulatory responses to exercise heat stress including: relative aerobic fitness (${\dot{V}}_{O_{2}\max}$; mL kg^−1^ min^−1^); body mass (kg); body surface area (m^2^); body fat (%); and age (Dervis et al., 2016). Metabolic heat production (MHP) per unit of body mass is generally regarded as the best way to standardise thermal strain in research studies (Cramer & Jay, 2016), but due to the occupational focus of the INM HIC, exercise intensity during the HTA is standardised by % ${\dot{V}}_{O_{2}\max}$. However, by matching groups for body mass and ${\dot{V}}_{O_{2}\max}$, it was also possible to effectively control for MHP at a group level. Although seasonal acclimatisation effects are minimal in temperate western climates (Bain & Jay, 2011), the majority of participants were tested outside of summer months (*n *= 23; 79% of each group) with an identical percentage of each group tested in the summer months (*n *= 6; 21% of each group).

Exclusion criteria for both groups included: diagnosed cardiovascular, metabolic or respiratory conditions (excluding asthma); recent blood donation (within 1 week of commencing study); inadequate understanding of English where no translator was available; current participation in any other research study which could influence their responses; GI diseases and/or disorders; adherence to any GI‐focused dietary regimes (such as low fermentable oligo‐, di‐, mono‐saccharides and polyols (FODMAP) or fibre‐modified diets) within the previous 3 months; consumption of potential modifiers of GI integrity (e.g., prebiotics, probiotics and/or antibiotics) within the previous 3 months; consumption of nonsteroidal anti‐inflammatory medications (NSAIDs) and/or stool altering medications (e.g., laxatives and anti‐diarrhoea) within 1 month of the experimental protocol.

### 2.4 Heat illness clinic routine procedures

Patient and matched control participants underwent identical procedures at the INM HIC, which have recently been described in detail (House et al., 2021). Briefly, participants arrived at 08.00 h, having been instructed to refrain from alcohol, caffeine‐containing drinks and maximal exercise 24 h before their visit and to arrive hydrated. All exercise testing was conducted in an environmental chamber (*T*_amb_ 34.7 ± 0.7°C; relative humidity 44.4 ± 3.1%) with other procedures conducted in an adjacent room (*T*_amb_ ∼21°C). Participants underwent an initial assessment where stature (Harpenden stadiometer, Holtain, Crymych, UK), body mass (Electronic Weight Indicator, Model I10, Ohaus Corporation, NJ, USA), blood pressure (M6, Omron, Milton Keynes, UK), resting 12 lead ECG (Mac 1600, GE Healthcare, Chicago, IL, USA), body composition (Bodystat 1500 MDD, Bodystat, Sulby, Isle of Man) and urine specific gravity (Multistix 10SG, Siemens, Munich, Germany) were assessed. Where urine specific gravity was >1.015 (Casa et al., 2000), participants were instructed to drink water to ensure hydration by the start of the HTA. They then underwent assessment of ${\dot{V}}_{O_{2}\max}$ on a motorised treadmill (Woodway PPS, Cranlea, Birmingham, UK). Participants started walking at a speed of 6 km h^−1^ (0% gradient), following which treadmill speed was increased 1 km h^−1^ per minute until reaching 13 km h^−1^. Thereafter, the treadmill gradient was increased by 2% per minute until the participant reached volitional exhaustion, which typically occurred within 8–15 min. Participants then rested for 45 min before being prepared for the HTA. Participants were encouraged to drink water to thirst during this period as drinking was not permitted during the HTA.

Prior to the HTA participants voided their bladder, body mass was measured and a calibrated thermistor (YSI 400 series, Smiths Medical Int Ltd, Luton, UK) was inserted to 10 cm beyond the anal sphincter for the measurement of rectal temperature (*T*_rec_). Four skin thermistors (Grant Instruments, Cambridge, UK) were instrumented on the chest, biceps, thigh and calf for the calculation of mean skin temperature (*T̅*_sk_) in accordance with Ramanathan (1964), with mean body temperature (*T̅*_b_) calculated as (0.9 × *T*_rec_) + (0.1 × *T̅*_sk_) (Sawka et al., 2011). Thereafter, participants donned sports trainers, standard British military issue multi‐terrain pattern fatigues including green static T‐shirt, long trousers and long‐sleeved jacket and entered the environmental chamber. The HTA was conducted on the motorised treadmill as described previously (House et al., 2021) and consisted of two phases as follows.

Phase 1 (0–30 min): participants carried a ∼14 kg rucksack and walked on the treadmill with the speed and gradient set to elicit a work intensity equivalent to 60% ${\dot{V}}_{O_{2}\max}$. This phase was intended to elicit an uncompensable heat stress.

Phase 2 (30–90 min): after 30 min the rucksack and jacket were removed. The T‐shirt was removed at minute 45 and the participant continued to walk on the treadmill until minute 60 and was stopped if a plateau (i.e., two identical consecutive 5‐min readings) or fall in *T*_rec_ occurred. If *T*_rec_ continued to rise, the participant continued to exercise until a plateau occurred, or 90 min elapsed. If *T*_rec_ reached 39.5°C at any point during the HTA the test was terminated and the participant was removed from the chamber and cooled; the HTA was also terminated on the request of the participant. This phase was intended to elicit a compensable heat stress. Individuals were classified as heat *tolerant* if at, or after, minute 60, but before minute 90, a plateau or fall in *T*_rec_ occurred. Participants were classified as heat intolerant if no plateau in *T*_rec_ occurred, *T*_rec_ reached ≥ 39.5°C, or the HTA was terminated by the participant or medical officer.

In addition to termination values, Phase 1 and Phase 2 data were reported as the rate of rise per unit of time for thermophysiological data or the delta change (Δ) for perceptual data. During the HTA expired gases were measured at minutes 5–7, 35–37 and 50–52 to verify the intensity of exercise, with MHP calculated from metabolic energy expenditure − external work (calculated incorporating treadmill speed, gradient and mass) according to Cramer and Jay (2019). Upon termination of the HTA, a post‐HTA body weight was obtained which was used in conjunction with the pre‐HTA mass to calculate whole body sweat rate (WBSR) relative to body surface area (Cramer & Jay, 2016).

### 2.5 Additional measures

**FIGURE 1:**
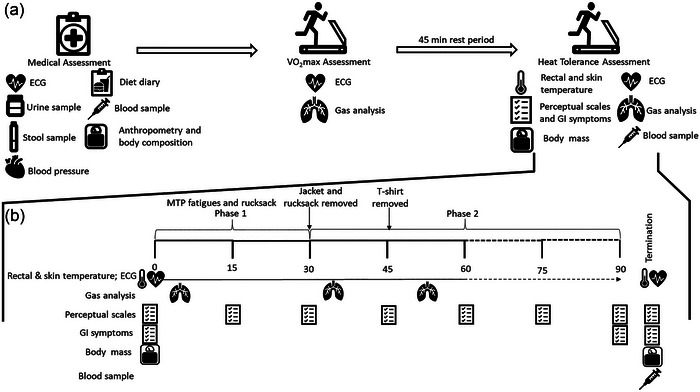
(a) Schematic overview of the Institute of Naval Medicine's Heat Illness Clinic, comprising: (i) medical assessment, (ii) ${\dot{V}}_{O_{2}\max}$ assessment, and (iii) HTA. (b) Detailed overview of the HTA. ECG, electrocardiogram; GI, gastrointestinal; HTA, heat tolerance assessment; MTP, multi‐terrain pattern.

In addition to the standard procedures of the INM HIC, a number of additional measures were obtained to support the study aims (summarised in Figure 1).

#### 2.5.1 Nutritional data

Participants completed a 3‐day standardised format dietary log prior to attending the HIC, which included detailed descriptions of the types and amount of food and drink consumed. Diet is known to influence the GI microbiota (Shanahan et al., 2021) and a 3‐day diet log is sufficient to capture participant diet characteristics (Johnson et al., 2020). The dietary log was analysed for macronutrient intake (energy (kcal), protein (g), carbohydrate (g) and fat (g)) using the Cronometer app.

#### 2.5.2 Urine analysis

An aliquot of urine was collected from the routine urine sample (prior to dipstick analysis) into a microcentrifuge tube (1.5 mL tube, Sarstedt, Nümbrecht, Germany) and stored at −20°C (World Health Organization, 2002). Subsequently, urine samples were left to thaw, and urine osmolality measured by freezing point depression osmometry (Gonotec, Berlin, Germany; coefficient of variation (CV) = 0.7%). Samples were measured in duplicate if their range was ≤2 mOsm kg^−1^ between 0 and 400 mOsm kg^−1^, or ≤4 mOsm kg^−1^ at >400 mOsm kg^−1^. If duplicates were outside of these values, a third measurement was taken.

#### 2.5.3 Perceptual scales

Rating of perceived exertion (RPE; Borg, 1982) and thermal comfort (TC) and thermal sensation (TS) using a modified Gagge et al. (1969) scale, as described by Borg et al. (2017), were assessed every 15 min during the HTA. A modified version of the visual analogue GI symptoms (GIS) scale (Gaskell et al., 2019) was used to assess GIS pre‐HTA and immediately after completion of the HTA. Participants were briefed on each scale prior to usage and they verbally confirmed their understanding.

#### 2.5.4 Blood analysis

Blood samples were obtained at baseline (after completion of medical assessment) and within 15 min of HTA termination in a seated position. Venous blood was taken from the antecubital vein using a 21‐gauge needle (Becton Dickinson, USA) and collected into serum (6.0 mL; Becton Dickinson, Franklin Lakes, NJ, USA) and EDTA (10 mL; Becton Dickinson) vacutainers. A proportion of the whole blood from the EDTA vacutainer was removed to allow the measurement of haemoglobin concentration (CV 1.3%) and haematocrit (CV 0.9%) to determine the change in plasma volume (Dill & Costill, 1974). Haemoglobin was measured in triplicate from 10 µL Microcuvettes (Hemocue, Ängelholm, Sweden) using a Hemocue analyser (Hb301). Haematocrit was measured in triplicate from haematocrit tubes (Hawksley & Sons Ltd, Lancing, UK), centrifuged for 8 min at 6.5 *g* (iFuge, Gandhinagar, India), using a micro‐haematocrit reader (Hawksley & Sons Ltd).

A centrifuge (Labofuge 400r, Heraeus, Hanau, Germany) was used (1500 *g* for 10 min at 4°C) to separate the plasma or serum from the cells in the remainder of the sample; serum vacutainers were left to clot for 1 h before being centrifuged. Aliquots (0.5 mL) of plasma and serum were then stored in microcentrifuge tubes (1.5 mL tube, Sarstedt) using sterile transfer pipettes (Sarstedt) and frozen at −80°C for subsequent biochemical analyses.

Serum intestinal fatty acid binding protein concentration ([I‐FABP]; DY990, R&D Systems, USA; dilution 1:5; intra‐assay CV 3.6%), plasma claudin 3 concentration ([CLDN‐3]; NBP2‐75328, Novus Biologicals, Littleton, CO, USA; neat; intra‐assay CV 6.1%), plasma lipopolysaccharide binding protein concentration ([LBP]; DY870‐05, R&D Systems, Minneapolis, MN, USA, USA; dilution 1:800; intra‐assay CV 3.0%), plasma soluble cluster of differentiation 14 concentration ([sCD14]; DY870‐05, R&D Systems; dilution 1:800; intra‐assay CV 3.4%) and plasma interleukin 6 concentration ([IL‐6]; HS600C, R&D Systems; neat; intra‐assay CV 3.4%) were analysed in duplicate using commercially available enzyme‐linked immunosorbent assay kits and quantified using a plate reader (MRX, Dynex Technologies, Chantilly, VA, USA). Serum C‐reactive protein concentration ([CRP]) was analysed on a Cobas 701 analyser (Roche, Burgess Hill, UK) using the Tina‐quant C‐Reactive Protein IV kit with values <1 mg L^−1^ coded as 0 in analysis. Post‐HTA blood biomarker concentrations were adjusted for plasma volume changes as described previously (Dill & Costill, 1974).

#### 2.5.5 Stool samples

Stool samples were collected for assessment of the faecal microbiome composition. Samples were provided on the morning of the HIC; a stool collection device (Abbexa, Cambridge, UK) was placed over the toilet seat and a pea‐sized sample, free from urine, was transferred into the sterile collection kit (80.734.001, Sarstedt). Upon receipt, stool samples were placed into a −80°C freezer (time at room temperature: 48 ± 49 min). Stool samples were subsequently transported from the INM to the University of Portsmouth (30‐min journey) in a specialised insulated medical freezer box with the panels frozen to −80°C and placed into the −80°C freezer until DNA extraction.

### 2.6 Bacterial DNA extraction

DNA was extracted from stool samples in batches with a positive (D6300, ZymoBIOMICS Microbial Community Standard, Irvine, CA, USA) and negative control (nuclease free water). Bacterial DNA was extracted using the QIAamp PowerFecal Pro DNA kit (51804, Qiagen, Hilden, Germany) according to the manufacturer's instructions. DNA was quantified by a spectrophotometer (DS‐11, DeNovix, Wilmington, DE, USA). The purified DNA was stored at −80°C until sequencing.

### 2.7 16S rRNA gene sequencing

Purified DNA was sent on dry ice to the National Oceanography Centre (NOC), University of Southampton for 16S rRNA gene amplicon sequencing. A PCR workflow was performed for library preparation using universal primers specific for the V3 and V4 hypervariable regions of the 16S rRNA gene (341F 5′‐CCTACGGGNGGCWGCAG‐3′ and 805R 5′‐GACTACHVGGGTATCTAATCC‐3′ (Klindworth et al., 2013)). PCR reactions were conducted on an Applied Biosystems Veriti 96 Well Thermal Cycler (Thermo Fisher Scientific, Waltham, MA, USA) using the following protocol: (i) initial denaturation for 3 min at 98°C, (ii) 25 cycles of denaturation for 30 s at 98°C, annealing for 30 s at 55°C, extension for 30 s at 72°C, (iii) final extension for 5 min at 72°C. AMPure XP beads (Beckman Coulter, Brea, CA, USA) were used to purify the 16S rRNA gene V3 and V4 amplicons away from free primers and primer dimer species. Individual libraries were indexed with eight cycles of PCR using Illumina (San Diego, CA, USA) DNA/RNA UD indexes and purified again using AMPure XP beads. Individual libraries were quantified using the Quant‐iT dsDNA High‐Sensitivity Assay Kit (Thermo Fisher Scientific) and quality controlled using the Agilent Bioanalyzer DNA1000 kit (Agilent Technologies, Santa Clara, CA, USA), prior to normalisation and pooling. Cluster generation and sequencing were conducted on an Illumina MiSeq benchtop sequencer using an Illumina MiSeq Reagent Kit v3 (600‐cycle).

### 2.8 Bioinfomatics

FastQC v0.11.9 (Andrews, 2012) was used to assess the quality of the raw read data to ensure the presence of no sequencing artefacts. Reads were trimmed to remove adapter sequences and poor quality reads using trim_galore v0.6.7 (Krueger, 2012) using parameters ‘‐q 20 –stringency 5 ‐e 0.1 –length 20 –trim‐n –clip_R1 10 –clip_R2 10 –phred33’. Potential host contamination was identified by mapping the trimmed reads against the GRCh38 human genome primary assembly from Ensemble (Cunningham et al., 2022) using Bowtie2 v2.5.0 (Langmead & Salzberg, 2012). Reads showing mapping to the human genome were identified using Samtools v1.15.1 and filtered from fastq files using seqkit v2.3.0 (Shen et al., 2016). Paired end reads were analysed to identify amplicon sequence variants (ASVs) using Quantitative Insights Into Microbial Ecology (QIIME2) v 2023.5.1 (Bolyen et al., 2019) using the DADA2 v1.26.0 (Callahan et al., 2016) denoising package in R v4.2.3 (R Core Team, 2023). ASVs were assigned taxonomy using a classifier trained using the REference Sequence annotation and CuRatIon (RESCRIPt) module in QIIME2 (Robeson et al., 2021). The classifier was trained using the SILVA v138.1 database (Quast et al., 2013), with sequences identified based on the 341F/805R primers used for PCR. The control and patient samples were rarefied to the lowest read count (33,102 reads) prior to further analysis.

### 2.9 Statistical analyses

Statistical analyses were conducted using the R programming language v4.3.1 (R Core Team, 2023) within RStudio (Posit team, 2023). The null hypothesis was rejected if *P* < 0.05. Participant characteristics, macronutrient intake, HTA variables, thermoregulatory and perceptual data were checked for normality using the Shapiro–Wilk test. Normally distributed data are presented as means ± SD and between‐group differences assessed using independent samples Student's *t*‐test; if the assumption of equal variances was violated, Welch's *t*‐test was used. If the assumption of normality was violated, data are presented as median (IQR), and differences assessed using the Mann–Whitney *U*‐test. Effect sizes are reported as Cohen's *d* (*d* < 0.2: trivial; >0.2: small >0.5: moderate; >0.8: large) and rank biserial correlation (*r* < 0.3: small; 0.3–0.5: medium; >0.5: large) for normally and non‐normally distributed data, respectively (Cohen, 2013; Kerby, 2014). Descriptives, normality tests, and independent *t*‐tests were calculated using the jamovi v2.3.4 package (Selker et al., 2022).

For analysis of repeated measure biomarkers (i.e., I‐FABP, CLDN3, LBP, sCD14, IL‐6), linear mixed‐effects models were used to compare biomarker concentration in controls and patients during the HTA using the lmerTest package v3.1‐3 (Kuznetsova et al., 2017). Group (control vs. patient), time point (pre vs. post) and their interaction were included as fixed effects to examine group differences. Additionally, each model included random intercepts assigned to each participant to account for within‐participant correlation for repeated measures. Model performance was assessed using the *performance* package v0.10.8 (Lüdecke et al., 2021). Data were log transformed where model residuals violated the assumption of normality. On one occasion, the residuals of the CLDN‐3 model as part of the heat tolerance sub‐analysis violated normality (*P* = 0.034). Multiple transformation attempts were performed (log, Box–Cox, and square root). However, all model residuals still violated normality. Linear mixed‐effects models are robust when assumptions are violated (Schielzeth et al., 2020). In the context of sub‐analysis, where we are underpowered (Brookes et al., 2004), we continued using the raw CLDN‐3 data as part of the sub‐analysis. The conditional (fixed and random effects) and marginal (fixed effects) *R* ^2^ values of each model are presented in each biomarker figure. Each model was run through a type III ANOVA (with Satterthwaite df) to determine significance of the fixed effects. Data were presented as estimated marginal means ± SE using the emmeans package v1.10.0 (Lenth, 2024). Partial eta squared was used to assess fixed effects (η^2^_p_: small >0.01; medium >0.06 and large >0.14) and calculated with the effectsize package v0.8.6 (Ben‐Shachar et al., 2020). Where biomarkers were only assessed at baseline (i.e., CRP) simple between‐group analyses were undertaken as described above.

For gut microbiome analysis, α‐diversity was assessed using: (i) observed diversity; (ii) Chao1 index (Chao, 1984); (iii) the Shannon index (Shannon, 1948); and (iv) the Simpson index (Simpson, 1949); these indices were assessed for normality and analysed for between‐group differences as described above. Principal coordinates analysis (PCoA) plots were produced based on the Bray–Curtis dissimilarity measure (Bray & Curtis, 1957) to assess β‐diversity. Analysis of α‐ and β‐diversity was performed using the phyloseq package v1.44.0 (McMurdie & Holmes, 2013). The effect of group (e.g., control vs. patient) on bacterial β‐diversity was assessed using permutational multivariate analysis of variance (PERMANOVA) analysis with the adonis2 function from the vegan v2.6‐4 package (Oksanen et al., 2022), using Bray–Curtis dissimilarity and 9999 permutations. The Firmicutes:Bacteroidota ratio and relative abundance of taxa at a phylum down to genus taxonomic level were compared between groups. For consistency, all relative abundance data was treated as non‐parametric and Mann–Whitney *U* comparisons of relative abundances were adjusted for multiple testing using the Benjamini and Hochberg (BH) correction (Benjamini & Hochberg, 1995). Differential abundance analysis was performed using the DESeq2 v1.36.0 (Love et al., 2014), ALDEx2 v1.34.0 (Fernandes et al., 2014), and ANCOMBC v2.4.0 packages (Lin & Peddada, 2020). A *P*‐value <0.05 was used to identify significantly different ASVs and adjusted *P*‐values (using BH correction) were used for all three methods. For DESeq2, the non‐rarefied feature tables were passed to the phyloseq_to_deseq2 function. Within the DESeq function, the test was set to ‘Wald’ and estimation of size factors set to ‘poscounts’ (Nearing et al., 2022). A fold change threshold of 2‐fold difference between the groups with an adjusted *P*‐value (adjusted for multiple testing using BH correction) was obtained. For ALDEx2, the non‐rarefied feature tables were passed to the aldex function which generated Monte Carlo samples of the Dirichlet distribution for each sample and converted each instance using a centred log‐ratio transform. Wilcoxon tests were performed on the transformed realisations, with the output returning the BH adjusted *P*‐values. For ANCOMBC, the non‐rarefied feature tables were passed to the ancombc function, with P_adj_method set to ‘BH’. The output returned BH adjusted *P*‐values. Counts of differentially abundant ASVs between all three packages were visualised with the ggvenn package v0.1.10 (Yan, 2023). ASVs that were differentially abundant in all three packages were considered significant (Nearing et al., 2022). Additional data processing was performed using the TidyVerse suite of packages v2.0.0 (Wickham et al., 2019) and all figures were plotted using the ggplot2 package v3.4.4 (Wickham, 2016); where (for clarity) *P*‐values are not displayed in figures, they can be located in the relevant text, table or Supporting information, as appropriate.

## 3 RESULTS

### 3.1 EHI patients and matched controls

**FIGURE 2:**
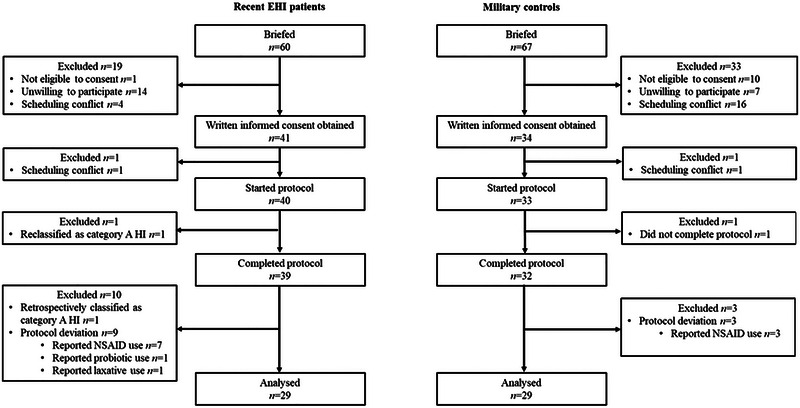
Consort flow diagram detailing participant flow through the study. EHI, exertional heat illness; HI, heat illness; NSAID, non‐steroidal anti‐inflammatory drug.

Participant flow through the study to achieve the target sample size is summarised in Figure 2.

Participant characteristics, macronutrient intake and HTA variables for Patients and Controls are presented in Table 1. For the recent EHI patients, the median time between the EHI incident and the HTA was 112 days (range: 83–238 days) and eight (28%) of these individuals reported a previous EHI to the one that instigated their HIC attendance. One individual in the recent EHI patient group terminated the HTA before completion of Phase 1 (for a non‐medical reason) and was excluded from analysis of the subsequent stage of the HTA and terminal indices. In addition, for a small number of participants skin thermistors became detached or equipment malfunctioned (e.g., heart rate monitor) in the thermally oppressive conditions; the ‘*n*’ for each variable is denoted in the relevant figure or table.

**TABLE 1:** Participant characteristics, 3‐day average macronutrient intake, and HTA variables for control participants without previous EHI history (Control; *n *= 29) and recent EHI patients (Patients; *n *= 29).

Variable	Control	Patient	*P*
Characteristics			
Age (years)	27 (8)	25 (8)	0.797
Height (cm)	181.6 ± 7.7	179.3 ± 7.0	0.251
Body mass (kg)	87.1 ± 9.4	86.1 ± 9.1	0.702
Body mass index (kg m^−2^)	26.5 (2.4)	27.0 (4.1)	0.431
Body fat percentage (%)	15.4 ± 3.6	15.3 ± 4.7	0.890
Body surface area (m^2^)	2.08 ± 0.14	2.05 ± 0.13	0.436
Mass specific surface area (cm^2^ kg^−1^)	240 ± 12	240 ± 13	0.861
${\dot{V}}_{O_{2}\max}$ (mL kg^−1^ min^−1^)	52.0 ± 5.3	50.5 ± 7.2	0.370
Urine osmolality (mOsm kg^−1^)	601 (684)	468 (577)	0.113
Macronutrient intake			
Energy (kcal day^−1^)	2480 ± 405	2379 ± 415	0.352
Carbohydrate (g day^−1^)	264.6 ± 61.0	247.0 ± 49.3	0.233
Fat (g day^−1^)	88.5 ± 20.2	94.9 ± 21.4	0.246
Protein (g day^−1^)	112.7 (17.7)	105.6 (34.9)	0.537
HTA variables			
Duration (min)	60 (5)	60 (5)	1.000
Phase 1 exercise intensity (% ${\dot{V}}_{O_{2}\max}$)	58.6 ± 2.0	59.3 ± 2.0	0.191
Phase 2 exercise intensity (% ${\dot{V}}_{O_{2}\max}$)	51.6 (3.4)	51.9 (3.4)^a^	0.722
Phase 1 MHP (W kg^−1^)	9.5 ± 0.9	9.3 ± 1.2	0.529
Phase 2 MHP (W kg^−1^)	8.4 (1.2)	8.3 (1.7)^a^	0.500

*Note*: Normally distributed data presented as means ± SD; non‐normally distributed data presented as median (IQR).

^a^ *n *= 28. Abbreviations: EHI, exertional heat illness; HTA, heat tolerance assessment; MHP, metabolic heat production; ${\dot{V}}_{O_{2}\max}$_,_ maximum rate of oxygen uptake.

#### 3.1.1 Thermophysiological responses during HTA

**FIGURE 3:**
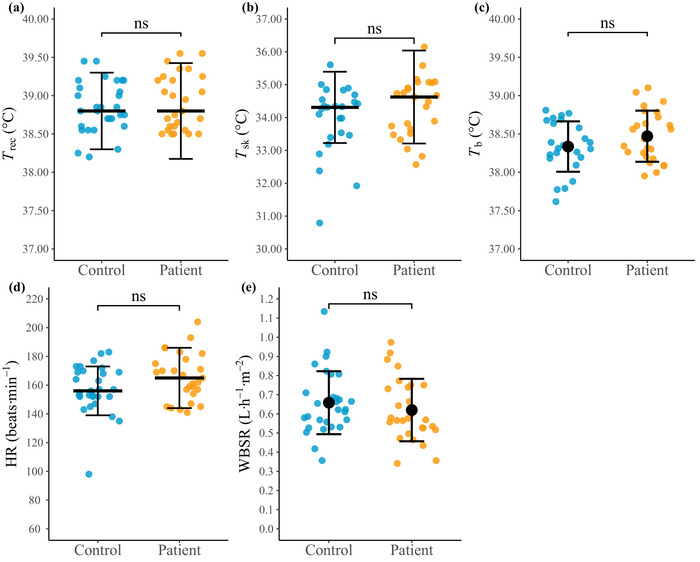
Terminal indices of thermoregulation and sweating during the HTA for control participants without previous EHI history (Control; *n *= 29) and recent EHI patients (Patient; *n *= 28). HTA termination values are presented for rectal temperature (*T*_rec_; a), mean skin temperature (*T*_sk_; b), mean body temperature (*T*_b_; c), heart rate (HR; d); WBSR is presented as the average during the HTA (WBSR; e). Normally distributed data presented as means ± SD (black circle); non‐normally distributed data resented as median (IQR) (wide black bar). (b, c) Control *n *= 25, Patient *n *= 25; (d) Control *n *= 29, Patient *n *= 27. ns, not significant, *P *> 0.05. EHI, exertional heat illness; HTA, heat tolerance assessment; WBSR, whole body sweat rate.

Terminal values for individual thermophysiological responses during the HTA are shown in Figure 3, with summary statistics for both groups (including Phase 1 and 2 values) presented in Supporting information, Table S1. There were no significant between‐group differences in the thermophysiological responses at termination or during the HTA, with the exception of recent EHI patients having a lower rate of rise in heart rate during Phase 1 (156 (32) versus 139 (36) beats min^−1^ h^−1^, *P* = 0.034, *r* = 0.33) and smaller reduction in the rate of rise in *T̅*_sk_ during Phase 2 (−3.37 (1.62) versus −1.85 (2.00)°C h^−1^, *P* = 0.003, *r* = 0.48) compared to matched controls.

#### 3.1.2 Perceptual responses during HTA

There were no significant differences in RPE, TC and TS between matched controls and patients (Supporting information, Figure S1 and Table S2). Similarly, there were no significant differences in GIS between matched controls and patients, with the exception of patients having a greater gut discomfort and greater lower GIS score post‐HTA (Supporting information, Table S3).

#### 3.1.3 GI microbiome

**FIGURE 4:**
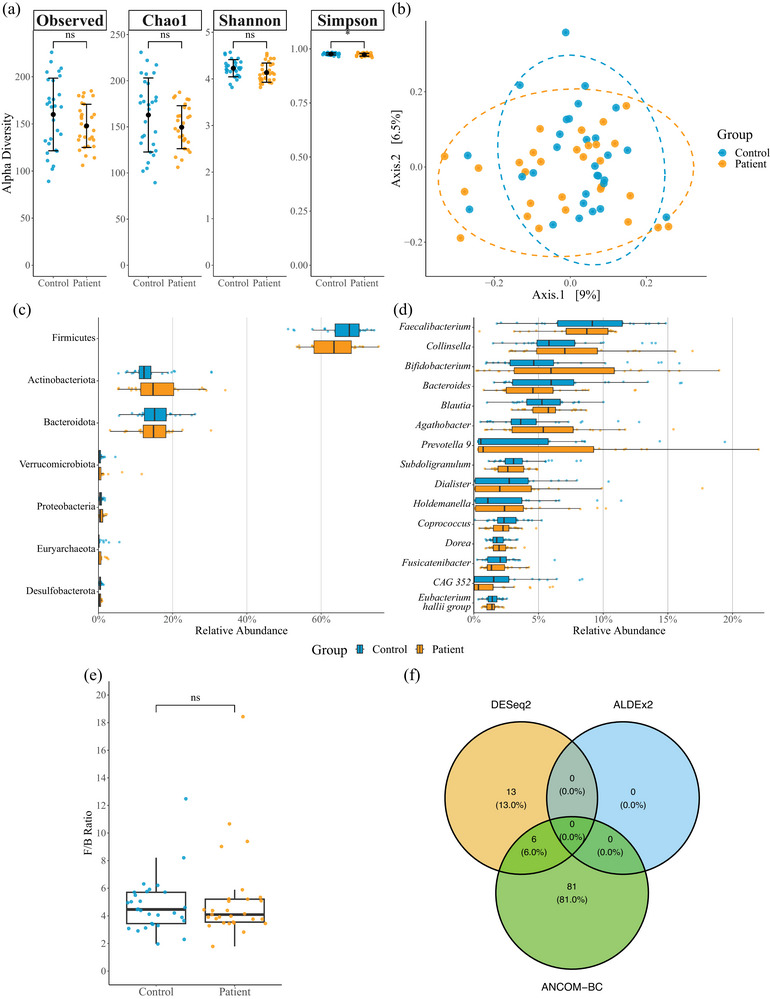
Comparison of faecal microbiome composition between control participants without previous EHI history (Control; *n *= 29) and recent EHI patients (Patient; *n *= 29). (a) Comparison of α‐diversity indices between controls and patients; from left to right: Observed, Chao1, Shannon, Simpson test of difference. (b) PCoA based on Bray–Curtis dissimilarity measure to assess β‐diversity. (c) Relative abundance of most common phyla compared between groups. (d) Relative abundance of most common genera compared between groups. (e) Firmicutes:Bacteroidota (F:B) ratio compared between groups. (f) Venn diagram comparing significant differentially abundant amplicon sequence variants between groups using three differential abundance methods (DESeq2, ALDEx2 and ANCOM‐BC). Mean ± SD, large black circle; median (IQR), wide black bar; ns, not significant, *P *> 0.05; *P < 0.05. EHI, exertional heat illness; PCoA, principal coordinate analysis.

The average number of unique taxa identified at each taxonomic level for controls and recent EHI patients is shown in Supporting information, Table S4. Observed (160 ± 39 vs. 148 ± 23, *P* = 0.154, *d* = 0.38), Chao1 (163 ± 40 vs. 150 ± 23, *P* = 0.129, *d* = 0.41) and Shannon indices of α‐diversity (4.23 ± 0.19 vs. 4.14 ± 0.21, *P* = 0.076, *d* = 0.47) were not different between matched controls and recent EHI patients, respectively (Figure 4a). However, the Simpson index of α‐diversity was higher in matched controls compared to patients (0.98 ± 0.00 vs. 0.97 ± 0.01, *P* = 0.030, *d* = 0.59). PERMANOVA identified no significant differences (*F*_(1,56)_ = 1.059, *P* = 0.313) in β‐diversity between controls and recent EHI patients (Figure 4b).

No between‐group differences were detected in the relative abundance of any taxa at the phylum level (most common phyla by relative abundance are presented in Figure 4c), or in the Firmicutes:Bacteroidota ratio between matched controls and recent EHI patients (4.5 (2.3) vs. 4.1 (1.7), *P =* 0.805, *r* = 0.04; Figure 4e). No between‐group differences were detected in the relative abundance of any taxa at the genus level (most common genera by relative abundance presented in Figure 4d), nor indeed at any other taxonomic level assessed (class, order, family). Relative abundance data at each taxonomic level are presented in Supporting information, Table S5.

DESeq2 and ANCOM‐BC analysis identified 19 and 87 (6 in common) differentially abundant ASVs, whereas ALDEx2 identified 0 differentially abundant ASVs (Figure 4f). Therefore, as there was no agreement for any ASV across all three methods, it was concluded that there were no differentially abundant ASVs between matched controls and patients.

#### 3.1.4 Biomarkers of GI integrity and inflammation

**FIGURE 5:**
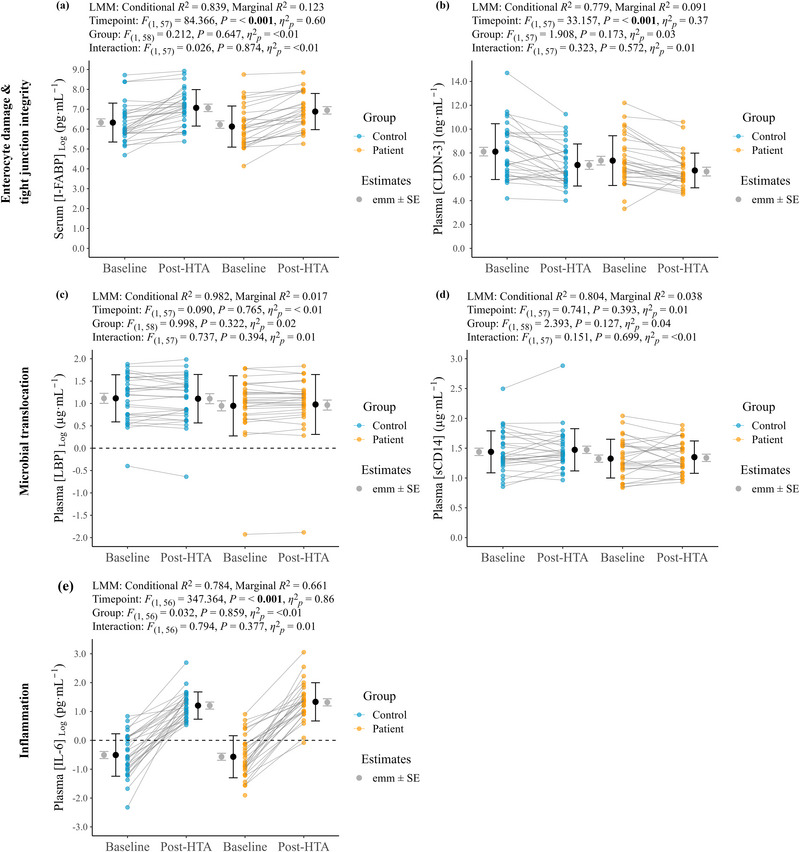
Repeated measure biomarkers of GI barrier integrity and inflammation at baseline and post‐HTA for control participants without previous EHI history (Control; *n *= 29) and recent EHI patients (Patients; *n *= 29). (a) Serum intestinal fatty acid binding protein concentration (I‐FABP). (b) Plasma claudin 3 concentration [CLDN‐3]. (c) Plasma liposaccharide binding protein concentration (LBP). (d) Plasma soluble Cluster of Differentiation 14 concentration (sCD14) (e) Plasma interleukin 6 concentration (IL‐6). Estimated marginal means (emm) ± SE (grey circle and error bar) are presented, alongside raw or log transformed mean ± SD (black circle and error bar), and individual paired data. EHI, exertional heat illness; GI, gastrointestinal; HTA, heat tolerance assessment.

No significant differences were evident between matched controls and patients for baseline serum (CRP) (0.00 (1.67) mg L^−1 ^vs. 0.00 (1.12) mg L^−1^, *P *= 0.437, *r* = 0.10). Pre–post changes in plasma volume were similar for matched controls (−1.5 ± 1.1%) and recent EHI patients (−1.4 ± 1.8%). Individual data for repeated‐measures biomarkers (i.e., baseline and post‐HTA) are shown in Figure 5, with summary data presented in Supporting information, Table S6 including raw (i.e., untransformed) data where log transformation was used. Significant time point effects were present for serum [I‐FABP], plasma [CLDN‐3] and plasma [IL‐6]. No significant main or interaction effects were present for plasma [LBP] and plasma [sCD14].

### 3.2 Heat tolerant and heat intolerant individuals

Six heat intolerant individuals were identified from the patient cohort, and six heat intolerant individuals were identified from the control cohort. Heat tolerant and heat intolerant individuals’ characteristics, macronutrient intake and HTA variables are presented in Table 2; with the exception of carbohydrate intake, these were not different between groups.

**TABLE 2:** Participant characteristics, 3‐day average macronutrient intake, and HTA variables for individuals classified as heat tolerant (*n *= 46) or heat intolerant (*n *= 12).

Variable	Heat tolerant	Heat intolerant	*P*
Characteristics			
Age (years)	27 (8)	24 (8)	0.218
Height (cm)	180.0 ± 7.1	182.2 ± 8.6	0.376
Body mass (kg)	85.9 ± 9.3	89.1 ± 8.6	0.295
Body mass index (kg m^−2^)	26.5 ± 2.7	26.8 ± 1.7	0.720
Body fat percentage (%)	15.0 ± 4.3	16.6 ± 3.5	0.256
Body surface area (m^2^)	2.05 (0.17)	2.08 (0.12)	0.387
Mass specific surface area (cm^2^ kg^−1^)	241 ± 13	237 ± 8	0.383
${\dot{V}}_{O_{2}\max}$ (mL kg^−1^ min^−1^)	51.6 ± 6.1	50.0 ± 7.2	0.437
Urine osmolality (mOsm kg^−1^)	505 (630)	540 (620)	0.977
Macronutrient intake			
Energy (kcal day^−1^)	2464 ± 423	2298 ± 337	0.215
Carbohydrate (g day^−1^)	263.9 ± 55.8	224.6 ± 44.8	0.028^a^
Fat (g day^−1^)	90.6 ± 21.1	95.6 ± 20.5	0.247
Protein (g day^−1^)	108.8 (25.9)	112.8 (16.3)	0.947
HTA variables			
Duration (min)	60 (5)	63 (29)	0.695
Phase 1 exercise intensity (%${\dot{V}}_{O_{2}\max}$)	58.8 ± 1.8	59.7 ± 2.6	0.293^a^
Phase 2 exercise intensity (%${\dot{V}}_{O_{2}\max}$)	51.5 ± 2.4	54.0 ± 4.1^b^	0.075^a^
Phase 1 MHP (W kg^−1^)	9.5 ± 1.1	9.3 ± 1.0	0.604
Phase 2 MHP (W kg^−1^)	8.4 (1.4)	8.4 (1.7)^b^	0.654

*Note*: Normally distributed data presented as means ± SD; non‐normally distributed data presented as median (IQR).

^a^ Welch's *t*‐test.

^b^ *n *= 11. *P < 0.05. Abbreviations: HTA, heat tolerance assessment; MHP, metabolic heat production; ${\dot{V}}_{O_{2}\max}$_,_ maximum rate of oxygen uptake.

#### 3.2.1 Thermophysiological responses during HTA

**FIGURE 6:**
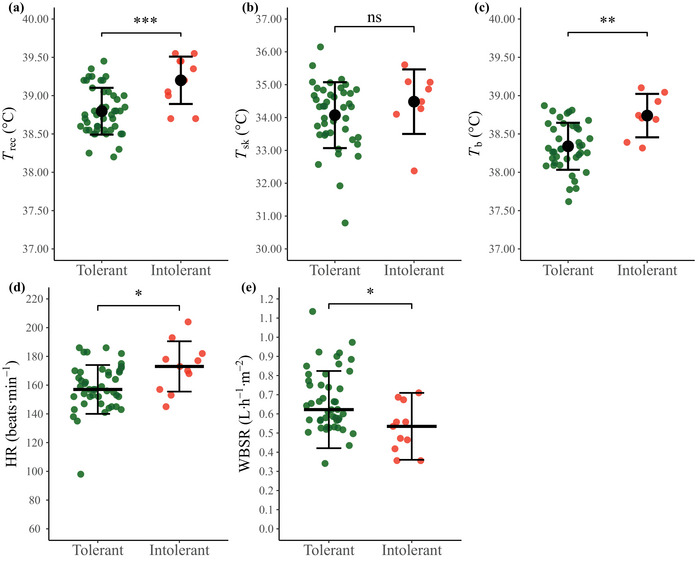
Terminal indices of thermoregulation and sweating during the HTA for heat tolerant (*n *= 46) and heat intolerant individuals (*n *= 11). HTA termination values are presented for rectal temperature (*T*_rec_; a), mean skin temperature (*T*_sk_; b), mean body temperature (*T*_b_; c), heart rate (HR; d); whole body sweat rate is presented as the average during the HTA (WBSR; e). Large black circle represents mean ± SD, large wide bar represents median (IQR). (b, c) tolerant *n *= 42, intolerant *n *= 8; (d) tolerant *n *= 45, intolerant *n *= 11. ns, not significant, *P *> 0.05; *P < 0.05; **P < 0.01; ***P < 0.001. HTA, heat tolerance assessment.

Terminal values for individual thermophysiological responses during the HTA, for heat tolerant and heat intolerant individuals, are presented in Figure 6 with summary statistics for both groups (including Phase 1 and 2 values) in Supporting information, Table S7. There were no between‐group differences in thermophysiological indices during Phase 1 of the HTA. However, during Phase 2, heat tolerant individuals had a smaller rate of rise in *T*_rec_ (tolerant = 0.70 (0.35) vs. intolerant = 1.20 (0.83)°C h^−1^, *P *< 0.001, *r *= 0.80), *T̅*_b_ (tolerant = 0.32 ± 0.28 vs. intolerant = 0.87 ± 0.32°C h^−1^, *P *< 0.001, *d* = −1.89) and heart rate (tolerant = −28 (25) vs. intolerant = −5 (21) beats min^−1^ h^−1^, *P *= 0.025, *r *= 0.44) than heat intolerant individuals, resulting in higher terminal values for these parameters in the heat intolerant individuals (*T*_rec_: tolerant = 38.80 ± 0.31 vs. intolerant = 39.20 ± 0.31°C, *P *< 0.001, *d* = −1.32; *T̅*_b_: tolerant = 38.34 ± 0.31 vs. intolerant = 38.74 ± 0.28°C, *P* = 0.001, *d* = −1.32; heart rate: tolerant = 157 (17) vs. intolerant = 173 (18) beats min^−1^, *P* = 0.020, *r* = 0.46). Heat intolerant individuals also had a lower WBSR during the HTA than heat tolerant individuals (tolerant = 0.62 (0.20) vs. intolerant = 0.53 (0.17) L m^−2^ h^−1^, *P* = 0.011, *r* = 0.49).

#### 3.2.2 Perceptual responses during HTA

Individual RPE, TC and TS during the HTA for heat tolerant and heat intolerant individuals is presented in Supporting information, Figure S2, with summary data in Supporting information, Table S8. Heat intolerant individuals reported a greater ΔRPE (−3.7 ± 2.0 vs. 0.5 ± 2.3, *P* < 0.001, *d* = −2.01) and ΔTC (−1.0 (0.5) vs. 0.5 (0.5), *P* < 0.001, *r* = 0.75) during Phase 2 as well as higher terminal values for these parameters (RPE: 10.0 (2.8) vs. 15.0 (2.0), *P* < 0.001, *r* = 0.75; TC: 2.0 (0.9) vs. 4.0 (0.8), *P* < 0.001, *r* = 0.85). Likewise, the ΔTS was higher in heat intolerant individuals during Phase 1 (2.0 (1.8) vs. 3.0 (2.0), *P* = 0.015, *r* = 0.45) and Phase 2 (−1.5 (1.0) vs. 0.0 (2.0), *P* = 0.001, *r* = 0.62), as well as at test termination (8.0 (1.0) vs. 11.0 (2.0), *P *< 0.001, *r* = 0.75). Summary data for GIS are shown in Supporting information, Table S9. Heat intolerant individuals reported greater dizziness pre‐HTA (0.0 (0.0) vs. 0.0 (0.3), *P* = 0.001, *r* = 0.25), and greater overall GIS (0.5 (3.0) vs. 8.0 (8.0), *P* < 0.001, *r* = 0.67), nausea (0.0 (0.0) vs. 3.0 (4.0), *P *< 0.001, *r* = 0.77), and dizziness (0.0 (1.0) vs. 4.0 (5.5), *P *< 0.001, *r* = 0.75) post‐HTA than heat tolerant individuals, but no other significant differences in GIS were observed.

#### 3.2.3 GI microbiome

**FIGURE 7:**
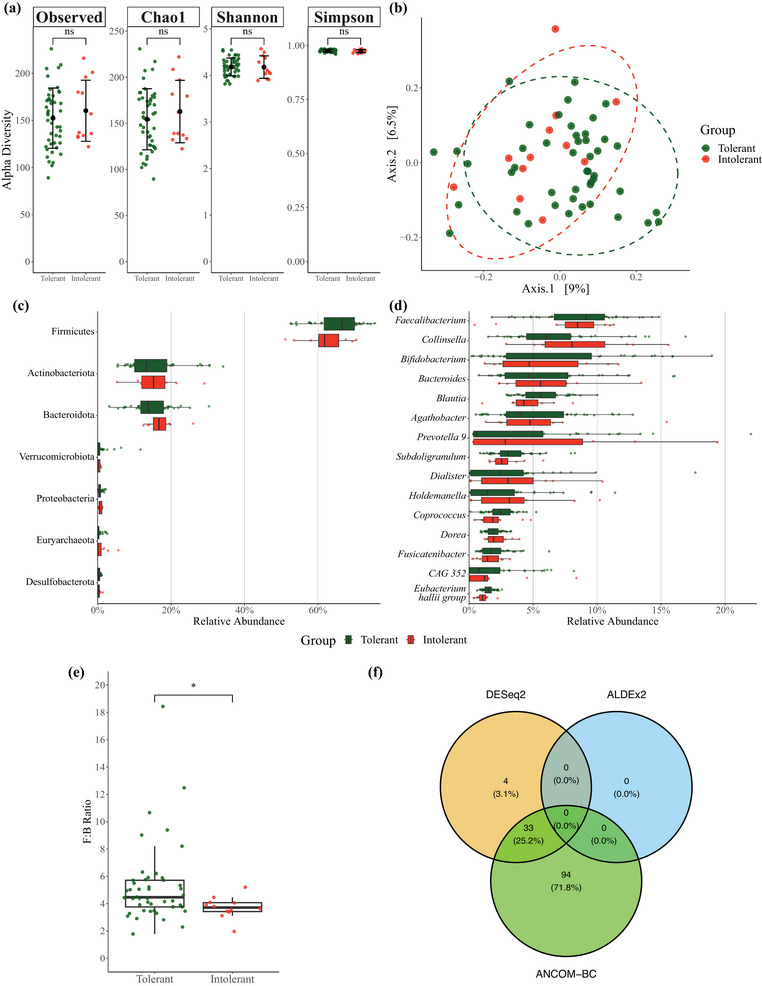
Comparison of faecal microbiome composition between heat tolerant (*n *= 46) and heat intolerant individuals (*n *= 12). (a) Comparison of α‐diversity indices between controls and patients; from left to right: Observed, Chao1, Shannon, Simpson. (b) PCoA based on Bray–Curtis dissimilarity measure to assess β‐diversity. (c) Relative abundance of most common phyla compared between groups. (d) Relative abundance of most common genera compared between groups. (e) Firmicutes:Bacteroidota (F:B) ratio compared between groups. (f) Venn diagram comparing significant differentially abundant amplicon sequence variants between groups using three differential abundance methods (DESeq2, ALDEx2 and ANCOM‐BC). ns, not significant, *P *> 0.05; *P < 0.05. Mean ± SD, large black circle; median (IQR), wide black bar. PCoA, principal coordinate analysis.

The average number of unique taxa identified at each taxonomic level for heat tolerant and heat intolerant individuals is shown in Supporting information, Table S10. Observed (152 ± 32 vs. 160 ± 33, *P* = 0.447, *d* = −0.25), Chao1 (154 ± 33 vs. 163 ± 34, *P* = 0.440, *d* = −0.25), Shannon (4.18 ± 0.19 vs. 4.18 ± 0.24, *P* = 0.931, *d* = 0.03) and Simpson (0.98 ± 0.01 vs. 0.97 ± 0.01, *P* = 0.413, *d* = 0.27) indices of α‐diversity were not different between heat tolerant and intolerant individuals, respectively (Figure 7a). PERMANOVA identified no difference (*F*_(1,56)_ = 1.052, *P* = 0.324) in β‐diversity between heat tolerant and intolerant individuals (Figure 7b).

No between‐group differences were detected in the relative abundance of any taxa at the phylum level (most common phyla presented in Figure 7c). However, heat tolerant individuals had a higher Firmicutes:Bacteroidota ratio than heat intolerant individuals (4.5 (2.0) vs. 3.7 (0.6), *P* = 0.019, *r* = 0.44; Figure 7e). No between‐group differences were detected in the relative abundance of any taxa at the genus level (most common genera presented in Figure 7d), nor indeed at any other taxonomic level assessed (class, order, family). Relative abundance data at each taxonomic level are presented in Supporting information, Table S11.

DESeq2 and ANCOM‐BC analysis identified 37 and 127 (33 in common) differentially abundant ASVs, whereas ALDEx2 identified 0 differentially abundant ASVs (Figure 7f). Therefore, as there was no agreement for any ASV across all three methods, it was concluded that there were no differentially abundant ASVs between heat tolerant and heat intolerant individuals.

#### 3.2.4 Biomarkers of GI integrity and inflammation

**FIGURE 8:**
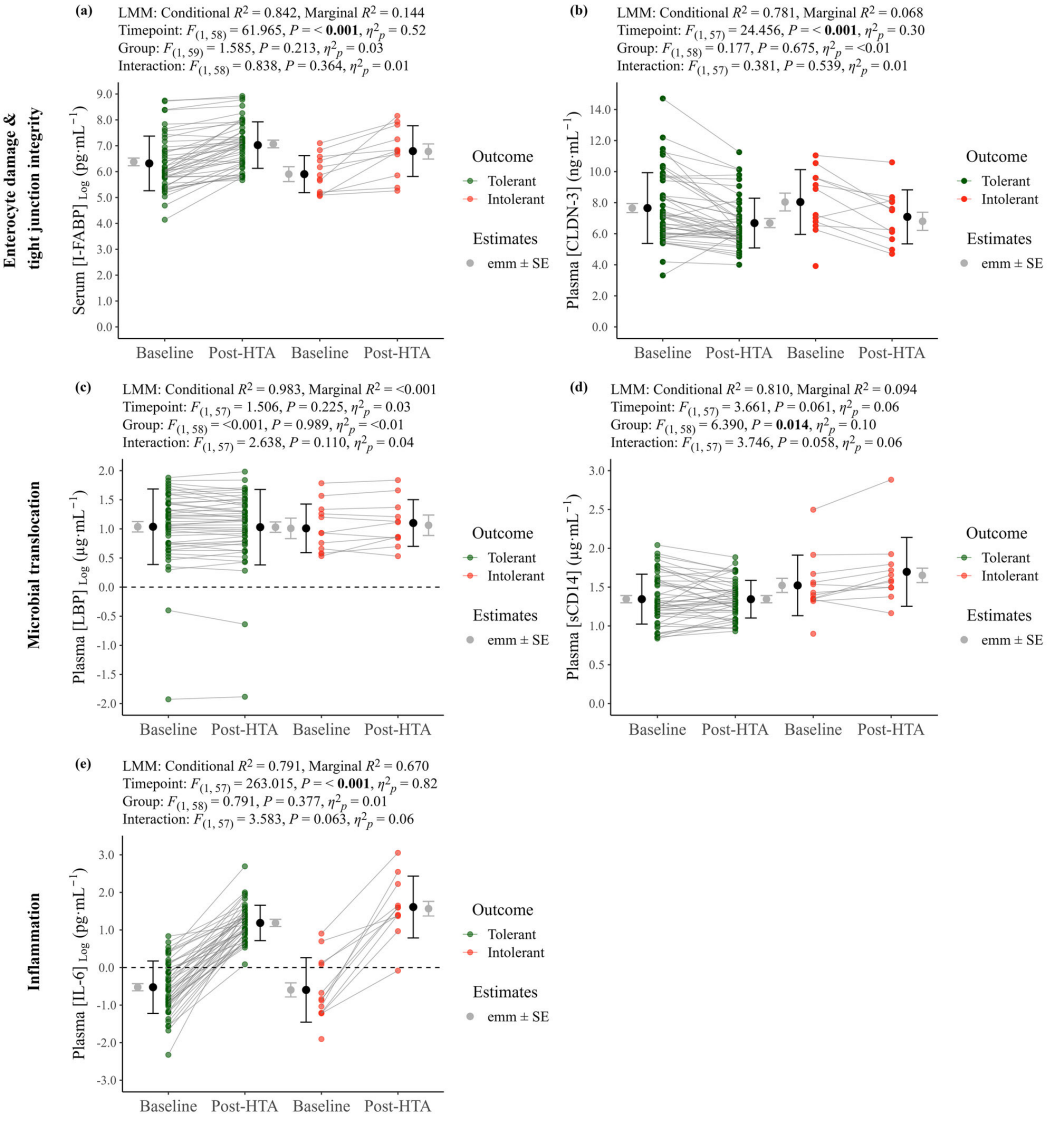
Biomarkers of GI integrity, GI damage and systemic inflammation at baseline and post‐HTA for heat tolerant (*n *= 46) and heat intolerant (*n *= 12) individuals. (a) Serum intestinal fatty acid binding protein concentration ([I‐FABP]). (b) Plasma claudin 3 concentration ([CLDN‐3]). (c) Plasma liposaccharide binding protein concentration ([LBP]). (d) Plasma soluble Cluster of Differentiation 14 concentration ([sCD14]). (e) Plasma interleukin 6 concentration ([IL‐6]). Estimated marginal means (emm) ± SE (grey circle and error bar) are presented, alongside raw or log transformed mean ± SD (black circle and error bar), and individual paired data. GI, gastrointestinal; HTA, heat tolerance assessment.

No significant differences were evident between heat tolerant and heat intolerant individuals for baseline serum [CRP] (0.00 (1.24) mg L^−1^ vs. 0.00 (1.66) mg L^−1^, *P* = 0.540, *r *= 0.10). Pre–post changes in plasma volume were similar for heat tolerant (−1.4% ± 1.6%) and heat intolerant (−1.4% ± 1.2%) individuals. Individual data for repeated‐measures biomarkers (i.e., baseline and post HTA) are shown in Figure 8, with summary data presented in Supporting information, Table S12 including raw (i.e., untransformed) data where log transformations were made. Significant time point effects were present for serum [I‐FABP], plasma [CLDN‐3] and plasma [IL‐6]. A significant group effect was present for plasma [sCD14]. No significant main or interaction effects were detected for plasma [LBP].

## 4 DISCUSSION

This is the first study to examine the faecal microbiome composition, and GI barrier integrity, inflammation and thermoregulation during exercise heat stress in individuals with a recent history of EHI, and to make comparisons with a matched cohort of individuals with no prior EHI history. We have also, for the first time, compared the composition of the faecal microbiome and indices of GI barrier integrity, inflammation and thermoregulation during exercise heat stress between individuals inferred to be heat tolerant and individuals inferred to be heat intolerant.

Our main findings were: (i) with the exception of the Simpson index, the detected α‐ and β‐diversity of the faecal microbiome did not differ between recent EHI patients and matched controls; (ii) the relative abundance of ASVs at phylum, class, order, family and genus taxonomic levels detected did not differ between recent EHI patients and matched controls, and no differentially abundant ASVs were identified between the groups; (iii) changes in GI barrier integrity and systemic inflammation in response to exercise heat stress were not different between recent EHI patients and matched controls; and (iv) the terminal thermoregulatory indices during the HTA did not differ between recent EHI patients and matched controls, but a subset of individuals across both groups demonstrated impaired heat tolerance, possibly due to a reduced sweating rate. Thereafter, comparison of heat tolerant and heat intolerant individuals demonstrated that: (v) the diversity and relative abundance of the faecal microbiome did not differ between heat tolerant and heat intolerant individuals, with the exception of a lower Firmicutes:Bacteroidota ratio in heat intolerant individuals; and (vi) changes in GI barrier integrity and systemic inflammation in response to exercise heat stress were not different between heat tolerant and heat intolerant individuals, with the exception that heat intolerant individuals had a higher plasma [sCD14].

### 4.1 GI microbial diversity and relative abundance in individuals with a recent EHI and matched controls

The GI microbiome has been hypothesised to play a role in severe EHI aetiology (Armstrong et al., 2012; Armstrong *et al.*, 2018; Roberts et al., 2021), but relevant empirical human studies are lacking. As such, we examined the composition of the faecal microbiome in humans with a recent EHI – a cohort at a substantially elevated risk of future EHI occurrence (Nelson et al., 2018a; Nelson et al., 2018b; Stearns et al., 2020) – and made comparison to a military control cohort matched for potential confounders (e.g., age, sex, dietary macronutrient intake, fitness and anthropometric factors; Clarke et al., 2014; Dervis et al., 2016; Estaki et al., 2016). With these strong controls in place, our data showed that, with the exception of the Simpson index, indices of α‐ and β‐diversity were not different between the cohorts. Indeed, whilst recent EHI patients had a lower Simpson index compared to controls, we are cautious in attribution of practical significance to this difference given the high numerical similarity between the groups. Previous research has reported 22 phyla and 113 genera in elite athletes, wheras separate high or low body mass index, age, and sex matched control groups had 9 or 11 phyla and 61 or 64 genera, respectively (Clarke et al., 2014). In the present study, ASVs corresponding to an average of six phyla (in both controls and patients), and 62 (control) or 58 (patient) genera were detected. Thus, the number of phyla and genera we detected in our cohorts were broadly in keeping with those previously reported for healthy male cohorts of a similar age to the present study. At both taxonomic levels the microbial diversity appears to be lower than that reported in elite athletes, and we cannot exclude the possibility that both our patients and controls have a sub‐optimal microbial biodiversity.

To provide greater insight into potential differences in GI microbial composition between our cohorts, we also compared the relative abundances of taxa from a phylum to genus level. After correcting for false discovery rate, no significant differences were identified between recent EHI patients and controls for the relative abundance of any taxa at any taxonomic level. Finally, we examined potential between‐group differences at the ASV level. Utilising a robust comparison method requiring agreement across multiple differential abundance methods (DESeq2, ALDEx2 and ANCOM‐BC), as has been recently recommended (Nearing et al., 2022), we did not identify any differentially abundant ASVs between our cohort of recent EHI patients and matched control participants. Taken together, our data indicate that substantial large‐scale diversity differences in the composition of the faecal microbiome were not evident between recent EHI patients and controls and suggest that differences in the composition of the faecal microbiome are unlikely to play a significant role in the elevated EHI susceptibility amongst recent EHI patients. Thus, we partially reject our first hypothesis. Additionally, given our strong matching approach, these data suggest that a recent EHI has limited effect on the faecal microbiome, but pre‐ and post‐EHI data are needed to verify this assertion.

### 4.2 GI barrier integrity and systemic inflammation in response to exercise heat stress in individuals with a recent EHI and matched controls

Resting serum [CRP] data indicated that there was no difference in baseline inflammation between our controls and recent EHI patients. However, the HTA elicited an increase in serum I‐FABP, a marker of enterocyte damage (Wells et al., 2017) that is related to GI barrier injury (Schellekens et al., 2014) and inflammation, as indicated by plasma [IL‐6] (i.e., main effect of ‘time’). However, no group or interaction effects were evident, and the magnitude of increase was in keeping with that observed in healthy individuals after exercise heat‐stress (Fortes et al., 2013; Lee et al., 2022; March et al., 2019; Ogden, Fallowfield, Child, Davison, Fleming, Edinburgh et al., 2020; Snipe et al., 2018; Starkie et al., 2005), with the increased serum [I‐FABP] notably lower than that reported after an EHI episode (Walter et al., 2021).

Conversely, plasma [CLDN‐3] decreased during the HTA (i.e., main effect of ‘time’), whereas, no ‘main’ or ‘interaction’ effects were evident for plasma [LBP] or [sCD14]. The decreased plasma [CLDN‐3] (Control, baseline vs. post‐HTA: 8.11 ± 0.36 vs. 6.99 ± 0.36; Patient: 7.36 ± 0.36 vs. 6.44 ± 0.37 ng mL^−1^) is in contrast to Ogden, Fallowfield, Child, Davison, Fleming, Edinburgh et al. (2020) who reported modest increases (∼0.3 ng mL^−1^) after fixed intensity walking under heat‐stress, albeit over a longer duration than the present study (80 min vs. 60 min), whereas the absence of effect of exercise heat stress on plasma [LBP] is in keeping with their data. Other studies using protocols with a greater exercise intensity (Lee et al., 2022; Wallett et al., 2021) and longer duration (Selkirk et al., 2008) have observed increases in circulating [LBP] and [sCD14] (Lee et al., 2022), potentially due to the high thermal strain achieved (e.g., peak *T*_rec_ ∼39.5°C; Lee et al., 2022; Wallett et al., 2021) or the low training status of participants (Selkirk et al., 2008). Together, our data indicate that enterocyte damage, tight junction integrity, microbial translocation and inflammation do not differ between recent EHI patients and controls, either at rest or in response to a sub‐clinical exercise heat stress.

### 4.3 Thermoregulatory responses to exercise heat stress in individuals with a recent EHI and matched controls

Our recent EHI patients and matched military controls demonstrated broadly similar thermoregulatory responses to the HTA. None of the terminal thermoregulatory variables differed between groups, which likely stems from the group similarities in key physiological and anthropometric factors affecting thermoregulation (Dervis et al., 2016). However, during Phase 1 of the HTA the rate of rise in heart rate was less in the patient group than in the control group, but further analysis indicated that the absolute heart rate values at the end of Phase 1 did not differ between groups and this effect was driven by a small baseline difference prior to commencing the HTA. Likewise, during Phase 2 of the HTA, the recent EHI patients also had a reduced rate of rise in *T̅*_sk_, but this did not affect *T*_rec_, *T̅*_b_ (which is the regulated variable during heat stress; Ravanelli et al., 2021), or the terminal *T̅*_sk_. Skin temperature is known to influence TS, which plays an important role in behavioural thermoregulation (Flouris & Schlader, 2015), although during exercise this may be through secondary effects on TC and RPE (Schlader et al., 2011). However, none of these perceptual indices differed between recent EHI patients and the control group over the course of the HTA. Recent EHI patients did, however, report greater gut discomfort and lower GIS post‐HTA, but median values remained low, and this was not consistent with changes in the aforementioned biomarkers of GI integrity during the HTA. Together, these observations suggest that when previous EHI patients are assessed more than ∼4 months post‐EHI, there are minimal group‐level differences in thermoregulation compared to non‐EHI controls; we therefore reject our second hypothesis. However, across both of our groups we identified a subset of individuals who were inferred as heat intolerant (6 in each group, 21%).

### 4.4 Thermoregulatory responses to exercise heat stress in individuals inferred as heat tolerant and individuals inferred as heat intolerant

During the initial uncompensable phase of the HTA (i.e., Phase 1), there were no differences in the rate of rise in any thermoregulatory variables between heat tolerant and heat intolerant individuals. However, during Phase 2 of the HTA, heat tolerant individuals had a greater reduction in cardiovascular strain, a reduced rate of rise in *T*_rec_ and *T̅*_b_, and were ultimately able to achieve a plateau in deep‐body temperature. In contrast, heat intolerant individuals were unable to achieve a deep‐body temperature plateau, possibly due to their significantly lower WBSR and a resulting lower evaporative heat loss (Cramer & Jay, 2016), which led to a higher terminal *T*_rec_, *T̅*_b_ and heart rate. Our data are consistent with House et al. (2021) who also reported that, compared to heat tolerant individuals, heat intolerant individuals had a reduced WBSR and a greater *T*_rec_ and heart rate after 60 min of the HTA. Importantly, the greater thermophysiological strain experienced by heat intolerant individuals in the present study was well sensed, as evidenced by the higher TS, TC and RPE during the latter portion of the HTA, suggesting that these individuals were deriving relevant perceptual information that would support thermoregulatory behaviours that might prevent progression to EHI if the rise in *T*_rec_ was unabated (e.g., reduce exercise pace to lower MHP; Corbett et al., 2018). Given that the groups were similar in terms of demographic, anthropometric and fitness factors, hydration state and MHP during the HTA, the reason for the lower WBSR in the heat intolerant individuals is unclear. A low acclimatisation state does not appear likely given that a similar percentage of heat tolerant and intolerant individuals was tested outside of summer months (78% vs. 83%), but some medical conditions are characterised by impaired sweating (e.g., ectodermal dysplasia; Massey et al., 2019) and it has been suggested that sweat gland density is influenced by childhood climate (Best et al., 2019); future studies should examine the mechanistic basis for this apparent hypohidrosis in heat intolerant individuals.

### 4.5 GI microbial diversity and relative abundance in heat tolerant and heat intolerant individuals

It has been suggested that the composition of the gut microbiota might influence thermoregulation during exercise heat stress (Bennett et al., 2020). However, similar to our primary analysis, indices of α‐diversity and β‐diversity were not different between heat tolerant and heat intolerant individuals, indicating levels of within‐ and between‐sample diversity did not differ across the groups. Nevertheless, heat intolerant individuals did have a significantly lower Firmicutes:Bacteroidota ratio than heat tolerant individuals. The Firmicutes:Bacteroidota ratio has been proposed as a putative marker of GI health (Magne et al., 2020), although increased values have been associated with the development of obesity, and conversely, decreased values have been associated with the development of inflammatory bowel disease (Stojanov et al., 2020). Some caution is warranted as our heat intolerant individuals had a reduced carbohydrate intake compared to tolerant individuals, and diet can affect the Firmicutes:Bacteroidota ratio (Beam et al., 2021). However, recent data did not identify a relationship between carbohydrate intake and the Firmicutes:Bacteroidota ratio, albeit in a different experimental context (Song et al., 2023). Moreover, it is important to note that neither of the individual relative abundances of Firmicutes or Bacteroidota, nor the relative abundances of all other identified taxa, or the differential abundance analysis of ASVs, differed between the heat tolerant and heat intolerant groups. Accordingly, we partially reject our third hypothesis.

### 4.6 GI barrier integrity and systemic inflammatory responses to exercise heat stress in heat tolerant and heat intolerant individuals

There were no baseline differences between the heat tolerant and heat intolerant groups for any of our biomarkers of GI barrier integrity, microbial translocation or systemic inflammation. Moreover, similar time point changes in serum [I‐FABP], plasma [CLDN‐3] and plasma [IL‐6] were observed over the course of the HTA. However, a significant group difference was evident for plasma [sCD14], albeit without an interaction, although both the ‘interaction’ and ‘time point’ effect for plasma [sCD14] displayed a medium effect size (η^2^_p_ = 0.06), with *P *< 0.10. Whilst we acknowledge that this comparison had lower power compared to our primary cohort comparison, it is plausible that with a greater sample size we may have identified a significant ‘interaction’ and ‘time point’ effect at the observed effect size. Additionally, whilst speculative, if these individuals continued exercise it is possible that they could have developed more pronounced microbial translocation precipitating the inflammatory responses that occur in the more severe forms of EHI (Garcia et al., 2022; Ogden, Child et al., 2020). Thus, we partially reject our fourth hypothesis.

### 4.7 Limitations

Bacterial DNA extracted from stool samples was used to provide an indication of the composition of the GI microbiota. Whilst this non‐invasive approach is commonly used, there may be differences between the stool derived microbiome composition and other approaches (e.g., biopsy; Nowicki et al., 2023). The 16S rRNA gene sequencing approach that we used enabled us to investigate, for the first time, the composition of the faecal microbiome from a phylum to genus taxonomic level, in recent EHI patients. However, metagenomics approaches provide higher taxonomic resolution and enable examination of the functional potential of the microbiome. Nevertheless, our contemporaneous measurements of systemic biomarkers of GI barrier integrity and damage, as well as microbial translocation and inflammation, provide valuable additional functional information during conditions of exercise heat stress. The degree of thermal strain that was elicited during the HTA was less than that which is associated with development of EHI and resulted in minimal microbial translocation and enterocyte damage, but the achievement of higher deep body temperatures would have been unethical in a cohort likely to be at increased risk of EHI, and the thermal stress was sufficient to distinguish between heat tolerant and heat intolerant individuals. Importantly, our laboratory model was similar in terms of exercise duration, intensity and activity type to that which caused the initial EHI for a number of our EHI patient group, but was often performed under more strenuous ambient conditions than those eliciting the initial incident. By enacting our strong controls, we have attempted to isolate our independent variable (recent EHI status); however, known EHI risk factors (e.g., fitness and high body mass) that we controlled for may also influence the GI microbiota, and we cannot exclude the possibility that these factors could influence EHI risk through pathways related to an altered GI microbiota. In addition, our primary aim was to compare the composition of the faecal microbiome between recent EHI patients and matched controls, and we cannot exclude the possibility that our secondary comparison of heat tolerant and heat intolerant individuals was underpowered to detect differences, and therefore this should be considered an exploratory analysis. Finally, our experimental design does not allow exclusion of the possibility that the EHI incident restored the faecal microbiome and/or strengthened the intestinal barrier of our EHI patients to a level comparable to the matched cohort, and our findings may not be relevant for females, given known sex differences in thermoregulation (Corbett et al., 2023). However, our study design does enable us to address the role of these factors on chronic EHI risk in males, in accordance with our experimental aim.

### 4.8 Conclusion

In conclusion, there were limited large‐scale differences in faecal microbiome composition, or the GI barrier integrity and inflammatory responses to exercise heat‐stress between recent EHI patients and matched controls, or between individuals classified as heat intolerant or heat tolerant. Together this suggests that: (i) large‐scale differences in faecal microbiome composition, and the thermoregulatory, GI barrier integrity and inflammatory responses to exercise heat stress are unlikely to contribute to the chronically elevated EHI risk in recent EHI patients; and (ii) heat intolerance does not appear to be the result of large‐scale differences in faecal microbiome composition, or the GI barrier integrity or inflammatory response to exercise heat stress.

## AUTHOR CONTRIBUTIONS

Alex A. M. Gould, Neil P. Walsh, Michael J. Tipton, Michael J. Zurawlew, Omar Tayari, Carol House, Simon K. Delves, Samuel C. Robson, Joy E. M. Watts, Andrew J. Roberts, Alex J. Rawcliffe, Megan R. Robinson, Jo Corbett conceived and designed research; Alex A. M. Gould, Omar Tayari, Carol House, performed experiments; Alex A. M. Gould, Samuel C. Robson, Janis J. Shute, Megan R. Robinson analysed data; Michael J. Tipton, Michael J. Zurawlew, Omar Tayari, Carol House, Simon K. Delves, Samuel C. Robson, Joy E. M. Watts, Andrew J. Roberts, Alex J. Rawcliffe, Megan R. Robinson, Jo Corbett interpreted the results of the experiments; Alex A. M. Gould, Samuel C. Robson prepared figures; Alex A. M. Gould, Samuel C. Robson, Joy E. M. Watts, Jo Corbett drafted the manuscript; Alex A. M. Gould, Neil P. Walsh, Michael J. Tipton, Michael J. Zurawlew, Omar Tayari, Carol House, Simon K. Delves, Samuel C. Robson, Joy E. M. Watts, Andrew J. Roberts, Alex J. Rawcliffe, Megan R. Robinson, Jo Corbett edited and revised the manuscript. All authors have read and approved the final version of this manuscript and agree to be accountable for all aspects of the work in ensuring that questions related to the accuracy or integrity of any part of the work are appropriately investigated and resolved. All persons designated as authors qualify for authorship, and all those who qualify for authorship are listed.

## CONFLICT OF INTEREST

None declared.

## Supporting information



Supplementary Tables S1–S12 and Supplementary Figures S1 and S2.

## Data Availability

Further information underpinning the findings reported in this paper has been made available as supplementary information. Additional data supporting the findings of this study are available from the corresponding author upon reasonable request and subsequent to the approval of the UK Ministry of Defence. The code used for the analysis of data in this manuscript are available under a GNU General Public License V3.0 at https://github.com/AlexG0uld/2024_Heat_Illness_Clinic_GI_Paradigm.
