# Supplementary material for: Faecal microbiome, gastrointestinal integrity, inflammation and thermoregulation in recent exertional heat illness patients and matched controls
Source: Exp Physiol. 2025 Jun 23;111(2):403–25. doi: 10.1113/EP092849 (PMC12857521; doi:10.1113/EP092849)
Supplement: Supplementary file 1 — Supplementary Tables S1–S12 and Supplementary Figures S1 and S2. [file EPH-111-403-s001.docx]

Supplementary Information

# Supplementary Tables

**Supplementary Table 1.** Thermophysiological responses during the Heat Tolerance Assessment for control participants without previous Exertional Heat Illness (EHI) history (Control: *n*=29) and recent EHI patients (Patient: *n*=28).

| **Variable** | **Control** | **Patient** | ***p* value** | **Effect size** |
| --- | --- | --- | --- | --- |
| **Rectal temperature** |  |  |  |  |
| Phase 1 (°C·h^-1^) | 1.90 (0.40) | 1.90 (0.40) | 0.968 | *r* < 0.01 |
| Phase 2 (°C·h^-1^) | 0.70 (0.38) | 0.80 (0.48) | 0.780 | *r* = 0.04 |
| Termination (°C) | 38.80 (0.50) | 38.80 (0.63) | 0.779 | *r* = 0.04 |
| **Mean skin temperature** |  |  |  |  |
| Phase 1 (°C·h^-1^) | 4.10 (2.40)^a^ | 4.12 (3.13)^a^ | 0.939 | *r* = 0.01 |
| Phase 2 (°C·h^-1^) | -3.37 (1.62)^a^ | -1.85 (2.00)^a^ | 0.003** | *r* = 0.48 |
| Termination (°C) | 34.31 (1.09)^a^ | 34.63 (1.42)^a^ | 0.165 | *r* = 0.23 |
| **Mean body temperature** |  |  |  |  |
| Phase 1 (°C·h^-1^) ^b^ | 2.16 ± 0.43^a^ | 2.12 ± 0.40^a^ | 0.704 | *d* = 0.11 |
| Phase 2 (°C·h^-1^) ^b^ | 0.33 ± 0.28^a^ | 0.49 ± 0.39^a^ | 0.116 | *d* = -0.45 |
| Termination (°C) ^b^ | 38.34 ± 0.33^a^ | 38.47 ± 0.33^a^ | 0.156 | *d* = -0.41 |
| **Heart rate** |  |  |  |  |
| Phase 1 (beats·min^-1^·h^-1^) | 156 (32) | 139 (36) | 0.034* | *r* = 0.33 |
| Phase 2 (beats·min^-1^·h^-1^) | -34 (25) | -22 (25) ^b^ | 0.093 | *r* = 0.26 |
| Termination (beats·min^-1^) | 156 (17) | 165 (21) ^b^ | 0.108 | *r* = 0.25 |
| **Sweat rate** |  |  |  |  |
| WBSR (L·m^-2^·h^-1^) | 0.66 ± 0.16 | 0.62 ± 0.16 | 0.379 | *d* = 0.23 |

Normally distributed data are presented as mean ± SD with between groups difference and effect size assessed by independent samples t-test and Cohen’s d, respectively. Non-normally distributed data are presented as median (IQR) with between groups difference and effect size assessed by Mann-Whitney U test and rank biserial correlation, respectively. WBSR=whole body sweat rate. ^a^ *n*=25; ^b^ *n*=27; *=*p*<0.05; **=*p*<0.01

**Supplementary Table 2.** Perceptual responses during the Heat Tolerance Assessment for control participants without previous Exertional Heat Illness (EHI) history (Control: *n*=29) and recent EHI patients (Patient: *n*=28).

| **Variable** | **Control** | **Patient** | ***p* value** | **Effect size** |
| --- | --- | --- | --- | --- |
| **RPE (scale: 6-20)** |  |  |  |  |
| Δ Phase 1 | 7.5 ± 1.8 | 6.5 ± 2.5 | 0.102 | *d* = 0.44 |
| Δ Phase 2 | -3.2 ± 2.7 | -2.6 ± 2.6 | 0.344 | *d* = -0.25 |
| Termination | 10.0 (3.0) | 10.0 (4.0) | 0.968 | *r* = 0.01 |
| **TC (scale 1.0-5.0)** |  |  |  |  |
| Δ Phase 1 | 1.5 (1.0) | 1.5 (1.0) | 0.668 | *r* = 0.07 |
| Δ Phase 2 | -0.7 ± 0.8 | -0.5 ± 0.7 | 0.412 | *d* = -0.22 |
| Termination | 2.5 (0.5) | 2.0 (1.5) | 0.314 | *r* = 0.15 |
| **TS (scale 5-13)** |  |  |  |  |
| Δ Phase 1 | 2.0 (1.0) | 2.0 (2.0) | 0.441 | *r* = 0.11 |
| Δ Phase 2 | -1.0 (1.0) | -1.0 (1.3) | 0.416 | *r* = 0.12 |
| Termination | 9.0 (1.0) | 8.0 (1.3) | 0.730 | *r* = 0.05 |

Normally distributed data are presented as mean ± SD with between groups difference and effect size assessed by independent samples t-test and Cohen’s d, respectively. Non-normally distributed data are presented as median (IQR) with between groups difference and effect size assessed by Mann-Whitney U test and rank biserial correlation, respectively. RPE=rating of perceived exertion; TC=thermal comfort; TS=thermal sensation.

**Supplementary Table 3.** Median (IQR) gastrointestinal symptoms pre- and post-Heat Tolerance Assessment for control participants without previous Exertional Heat Illness (EHI) history (Control: *n*=29) and recent EHI patients (Patient: *n*=29).

| **Variable** | **Control** | **Patient** | ***p* value** | **Effect size** |
| --- | --- | --- | --- | --- |
| **Pre-HTA GIS** |  |  |  |  |
| Gut discomfort | 0.0 (0.0) | 0.0 (0.0) | 0.081 | *r* = 0.10 |
| Overall GIS | 0.0 (0.0) | 0.0 (1.0) | 0.078 | *r* = 0.21 |
| Upper GIS | 0.0 (0.0) | 0.0 (0.0) | 1.000 | *r* < 0.01 |
| Lower GIS | 0.0 (0.0) | 0.0 (0.0) | 0.245 | *r* = 0.12 |
| Nausea | 0.0 (0.0) | 0.0 (0.0) | 0.334 | *r* = 0.03 |
| Dizziness | 0.0 (0.0) | 0.0 (0.0) | 0.081 | *r* = 0.10 |
| **Post-HTA GIS** |  |  |  |  |
| Gut discomfort | 0.0 (0.0) | 0.0 (1.0)^a^ | 0.003** | *r* = 0.31 |
| Overall GIS | 1.0 (2.0) | 3.0 (7.0)^a^ | 0.085 | *r* = 0.26 |
| Upper GIS | 0.0 (0.0) | 0.0 (1.0)^a^ | 0.161 | *r* = 0.16 |
| Lower GIS | 0.0 (0.0) | 0.0 (2.0)^a^ | 0.040* | *r* = 0.27 |
| Nausea | 0.0 (0.0) | 0.0 (0.3)^a^ | 0.446 | *r* = 0.08 |
| Dizziness | 0.0 (1.0) | 0.5 (2.0)^a^ | 0.297 | *r* = 0.15 |

Between groups difference and effect size assessed by Mann-Whitney U test and rank biserial correlation, respectively. GIS=gastrointestinal symptoms; HTA=heat tolerance assessment. ^a^ *n*=28. *=*p*<0.050; **=*p*<0.010.

**Supplementary Table 4.** Number of bacterial taxa detected at each taxonomic level for control participants without previous Exertional Heat Illness (EHI) history (Control: *n*=29) and recent EHI patients (Patient: *n*=29).

| **Group** | **Phyla** | **Classes** | **Orders** | **Families** | **Genera** | **ASVs** |
| --- | --- | --- | --- | --- | --- | --- |
| **Control** | 6 (1) | 9 (2) | 17 ± 2 | 30 ± 4 | 62 ± 9 | 160 ± 39 |
| **Patient** | 6 (2) | 9 (2) | 16 ± 2 | 29 ± 3 | 58 ± 7 | 148 ± 23 |

Normally distributed data are presented as mean ± SD. Non-normally distributed data are presented as median (IQR). ASV=amplicon sequence variant.

**Supplementary Table 5.** Median (IQR) relative abundance of bacterial taxa at a Phylum, Class, Order, Family, and Genus taxonomic level for control participants without previous Exertional Heat Illness (EHI) history (Control: *n*=29) and recent EHI patients (Patient: *n*=29).

| **Taxa** | **Control** | **Patient** | ***p* value** | ***p*_adj_ value** |
| --- | --- | --- | --- | --- |
| **Phylum** |  |  |  |  |
| Actinobacteriota | 12.3 (3.2) | 14.7 (8.9) | 0.227 | 0.681 |
| Bacteroidota | 15.1 (5.9) | 14.9 (6.1) | 0.762 | 0.870 |
| Cyanobacteria | 0.0 (0.0) | 0.0 (0.0) | 0.598 | 0.870 |
| Desulfobacterota | 0.5 (0.3) | 0.3 (0.5) | 0.173 | 0.681 |
| Euryarchaeota | 0.0 (0.2) | 0.0 (0.7) | 0.660 | 0.870 |
| Firmicutes | 67.6 (6.5) | 63.5 (10.1) | 0.082 | 0.681 |
| Proteobacteria | 0.5 (0.6) | 0.5 (0.9) | 0.773 | 0.870 |
| Spirochaetota | 0.0 (0.0) | 0.0 (0.0) | 0.334 | 0.752 |
| Verrucomicrobiota | 0.1 (0.5) | 0.1 (0.6) | 0.881 | 0.881 |
| **Class** |  |  |  |  |
| Actinobacteria | 4.6 (3.4) | 6.0 (7.7) | 0.338 | 0.633 |
| Alphaproteobacteria | 0.0 (0.0) | 0.0 (0.0) | 0.534 | 0.881 |
| Bacilli | 5.7 (5.4) | 5.8 (3.8) | 0.852 | 0.881 |
| Bacteroidia | 15.1 (5.9) | 14.9 (6.1) | 0.762 | 0.881 |
| Brachyspirae | 0.0 (0.0) | 0.0 (0.0) | 0.334 | 0.633 |
| Clostridia | 54.8 (9.9) | 51.1 (10.2) | 0.145 | 0.633 |
| Coriobacteriia | 7.7 (2.7) | 8.6 (4.1) | 0.307 | 0.633 |
| Desulfovibrionia | 0.5 (0.3) | 0.3 (0.5) | 0.173 | 0.633 |
| Gammaproteobacteria | 0.5 (0.5) | 0.5 (0.6) | 0.864 | 0.881 |
| Incertae_Sedis | 0.0 (0.0) | 0.0 (0.0) | 0.334 | 0.633 |
| Lentisphaeria | 0.0 (0.0) | 0.0 (0.0) | 0.161 | 0.633 |
| Methanobacteria | 0.0 (0.2) | 0.0 (0.7) | 0.660 | 0.881 |
| Negativicutes | 4.1 (4.6) | 4.3 (3.4) | 0.322 | 0.633 |
| Vampirivibrionia | 0.0 (0.0) | 0.0 (0.0) | 0.598 | 0.881 |
| Verrucomicrobiae | 0.1 (0.5) | 0.0 (0.6) | 0.881 | 0.881 |
| **Order** |  |  |  |  |
| Acidaminococcales | 0.3 (1.2) | 0.8 (1.9) | 0.096 | 0.619 |
| Actinomycetales | 0.0 (0.0) | 0.0 (0.0) | 0.958 | 0.967 |
| Bacteroidales | 15.1 (5.9) | 14.9 (6.1) | 0.762 | 0.898 |
| Bifidobacteriales | 4.6 (3.4) | 6.0 (7.7) | 0.338 | 0.619 |
| Brachyspirales | 0.0 (0.0) | 0.0 (0.0) | 0.334 | 0.619 |
| Burkholderiales | 0.2 (0.3) | 0.4 (0.4) | 0.311 | 0.619 |
| Christensenellales | 0.7 (0.8) | 0.7 (1.1) | 0.950 | 0.967 |
| Clostridia_UCG-014 | 0.5 (0.8) | 0.6 (0.7) | 0.561 | 0.771 |
| Clostridia_vadinBB60_group | 0.0 (0.0) | 0.0 (0.0) | 0.334 | 0.619 |
| Clostridiales | 1.0 (1.2) | 0.5 (0.8) | 0.046 | 0.406 |
| Coriobacteriales | 7.7 (2.7) | 8.6 (4.1) | 0.307 | 0.619 |
| Desulfovibrionales | 0.5 (0.3) | 0.3 (0.5) | 0.173 | 0.619 |
| DTU014 | 0.0 (0.0) | 0.0 (0.0) | 0.334 | 0.619 |
| Enterobacterales | 0.1 (0.2) | 0.1 (0.1) | 0.142 | 0.619 |
| Erysipelotrichales | 4.1 (5.2) | 5.1 (3.5) | 0.516 | 0.771 |
| Flavobacteriales | 0.0 (0.0) | 0.0 (0.0) | 0.334 | 0.619 |
| Gastranaerophilales | 0.0 (0.0) | 0.0 (0.0) | 0.598 | 0.790 |
| Izemoplasmatales | 0.0 (0.0) | 0.0 (0.0) | 0.543 | 0.771 |
| Lachnospirales | 28.1 (6.3) | 27.7 (6.6) | 0.939 | 0.967 |
| Lactobacillales | 0.7 (1.5) | 0.6 (0.7) | 0.419 | 0.727 |
| Methanobacteriales | 0.0 (0.2) | 0.0 (0.7) | 0.660 | 0.831 |
| Micrococcales | 0.0 (0.0) | 0.0 (0.0) | 0.334 | 0.619 |
| Monoglobales | 0.6 (0.5) | 0.3 (0.4) | 0.017 | 0.406 |
| Opitutales | 0.0 (0.0) | 0.0 (0.0) | 0.334 | 0.619 |
| Oscillospirales | 21.0 (4.0) | 18.2 (6.9) | 0.049 | 0.406 |
| Peptococcales | 0.0 (0.0) | 0.0 (0.0) | 0.680 | 0.831 |
| Peptostreptococcales-Tissierellales | 2.3 (2.6) | 1.5 (2.0) | 0.046 | 0.406 |
| RF39 | 0.1 (0.1) | 0.1 (0.1) | 0.470 | 0.771 |
| Rhodospirillales | 0.0 (0.0) | 0.0 (0.0) | 0.534 | 0.771 |
| Staphylococcales | 0.0 (0.0) | 0.0 (0.0) | 0.334 | 0.619 |
| Veillonellales-Selenomonadales | 3.3 (5.4) | 3.0 (4.3) | 0.925 | 0.967 |
| Verrucomicrobiales | 0.0 (0.5) | 0.0 (0.6) | 0.967 | 0.967 |
| Victivallales | 0.0 (0.0) | 0.0 (0.0) | 0.161 | 0.619 |
| **Family** |  |  |  |  |
| *[Clostridium]_methylpentosum_group* | 0.0 (0.0) | 0.0 (0.0) | 0.334 | 0.596 |
| *[Eubacterium]_coprostanoligenes_group* | 0.7 (0.7) | 0.7 (0.4) | 0.630 | 0.844 |
| *Acidaminococcaceae* | 0.3 (1.2) | 0.8 (1.9) | 0.096 | 0.596 |
| *Actinomycetaceae* | 0.0 (0.0) | 0.0 (0.0) | 0.958 | 0.983 |
| *Akkermansiaceae* | 0.0 (0.5) | 0.0 (0.6) | 0.967 | 0.983 |
| *Anaerovoracaceae* | 0.1 (0.1) | 0.1 (0.1) | 0.690 | 0.844 |
| *Atopobiaceae* | 0.1 (0.3) | 0.1 (0.4) | 0.630 | 0.844 |
| *Bacteroidaceae* | 6.0 (4.7) | 4.6 (3.6) | 0.301 | 0.596 |
| *Barnesiellaceae* | 0.7 (1.1) | 0.5 (0.5) | 0.179 | 0.596 |
| *Bifidobacteriaceae* | 4.6 (3.4) | 6.0 (7.7) | 0.338 | 0.596 |
| *Brachyspiraceae* | 0.0 (0.0) | 0.0 (0.0) | 0.334 | 0.596 |
| *Butyricicoccaceae* | 0.4 (0.4) | 0.6 (0.3) | 0.085 | 0.596 |
| *Christensenellaceae* | 0.7 (0.8) | 0.7 (1.1) | 0.950 | 0.983 |
| *Clostridia_UCG-014* | 0.5 (0.8) | 0.6 (0.7) | 0.561 | 0.844 |
| *Clostridia_vadinBB60_group* | 0.0 (0.0) | 0.0 (0.0) | 0.334 | 0.596 |
| *Clostridiaceae* | 1.0 (1.2) | 0.5 (0.8) | 0.046 | 0.596 |
| *Coriobacteriaceae* | 5.8 (2.9) | 7.0 (4.7) | 0.330 | 0.596 |
| *Coriobacteriales_Incertae_Sedis* | 0.0 (0.0) | 0.0 (0.0) | 0.839 | 0.932 |
| *Defluviitaleaceae* | 0.0 (0.0) | 0.0 (0.0) | 0.334 | 0.596 |
| *Desulfovibrionaceae* | 0.5 (0.3) | 0.3 (0.5) | 0.173 | 0.596 |
| *DTU014* | 0.0 (0.0) | 0.0 (0.0) | 0.334 | 0.596 |
| *Eggerthellaceae* | 1.2 (0.8) | 1.2 (0.9) | 0.732 | 0.854 |
| *Enterobacteriaceae* | 0.0 (0.1) | 0.0 (0.0) | 0.693 | 0.844 |
| *Enterococcaceae* | 0.0 (0.0) | 0.0 (0.0) | 1.000 | 1.000 |
| *Erysipelatoclostridiaceae* | 1.3 (2.2) | 1.9 (2.3) | 0.703 | 0.844 |
| *Erysipelotrichaceae* | 1.5 (3.5) | 2.9 (3.5) | 0.624 | 0.844 |
| *Family_XI* | 0.0 (0.0) | 0.0 (0.0) | 0.334 | 0.596 |
| *Flavobacteriaceae* | 0.0 (0.0) | 0.0 (0.0) | 0.334 | 0.596 |
| *Gastranaerophilales* | 0.0 (0.0) | 0.0 (0.0) | 0.598 | 0.844 |
| *Hungateiclostridiaceae* | 0.0 (0.0) | 0.0 (0.0) | 0.334 | 0.596 |
| *Izemoplasmatales* | 0.0 (0.0) | 0.0 (0.0) | 0.543 | 0.844 |
| *Lachnospiraceae* | 28.1 (6.3) | 27.7 (6.6) | 0.939 | 0.983 |
| *Lactobacillaceae* | 0.0 (0.0) | 0.0 (0.0) | 0.740 | 0.854 |
| *Marinifilaceae* | 0.4 (0.2) | 0.4 (0.3) | 0.901 | 0.983 |
| *Methanobacteriaceae* | 0.0 (0.2) | 0.0 (0.7) | 0.660 | 0.844 |
| *Micrococcaceae* | 0.0 (0.0) | 0.0 (0.0) | 0.334 | 0.596 |
| *Monoglobaceae* | 0.6 (0.5) | 0.3 (0.4) | 0.017 | 0.506 |
| *Muribaculaceae* | 0.0 (0.1) | 0.0 (0.0) | 0.088 | 0.596 |
| *Oscillospiraceae* | 1.7 (1.5) | 1.4 (1.4) | 0.164 | 0.596 |
| *Oxalobacteraceae* | 0.0 (0.0) | 0.0 (0.0) | 0.571 | 0.844 |
| *Pasteurellaceae* | 0.0 (0.1) | 0.0 (0.0) | 0.010 | 0.506 |
| *Peptococcaceae* | 0.0 (0.0) | 0.0 (0.0) | 0.680 | 0.844 |
| *Peptostreptococcaceae* | 1.9 (2.5) | 1.5 (2.0) | 0.049 | 0.596 |
| *Porphyromonadaceae* | 0.0 (0.0) | 0.0 (0.0) | 0.161 | 0.596 |
| *Prevotellaceae* | 2.2 (6.8) | 5.2 (10.0) | 0.272 | 0.596 |
| *Puniceicoccaceae* | 0.0 (0.0) | 0.0 (0.0) | 0.334 | 0.596 |
| *RF39* | 0.1 (0.1) | 0.1 (0.1) | 0.470 | 0.806 |
| *Rikenellaceae* | 1.0 (0.9) | 1.0 (0.9) | 0.331 | 0.596 |
| *Ruminococcaceae* | 17.6 (4.1) | 15.6 (5.5) | 0.064 | 0.596 |
| *Selenomonadaceae* | 0.0 (0.0) | 0.0 (0.0) | 0.656 | 0.844 |
| *Staphylococcaceae* | 0.0 (0.0) | 0.0 (0.0) | 0.334 | 0.596 |
| *Streptococcaceae* | 0.6 (1.4) | 0.5 (0.6) | 0.316 | 0.596 |
| *Succinivibrionaceae* | 0.0 (0.0) | 0.0 (0.0) | 0.161 | 0.596 |
| *Sutterellaceae* | 0.2 (0.3) | 0.4 (0.4) | 0.310 | 0.596 |
| *Tannerellaceae* | 0.9 (0.5) | 0.8 (0.5) | 0.293 | 0.596 |
| *UCG-010* | 0.1 (0.0) | 0.1 (0.0) | 0.540 | 0.844 |
| uncultured | 0.0 (0.1) | 0.0 (0.1) | 0.679 | 0.844 |
| *vadinBE97* | 0.0 (0.0) | 0.0 (0.0) | 0.334 | 0.596 |
| *Veillonellaceae* | 3.2 (4.1) | 2.4 (4.0) | 0.815 | 0.922 |
| *Victivallaceae* | 0.0 (0.0) | 0.0 (0.0) | 0.161 | 0.596 |
| **Genus** |  |  |  |  |
| *[Clostridium]_innocuum_group* | 0.0 (0.0) | 0.0 (0.0) | 0.571 | 0.803 |
| *[Clostridium]_methylpentosum_group* | 0.0 (0.0) | 0.0 (0.0) | 0.334 | 0.670 |
| *[Eubacterium]_brachy_group* | 0.0 (0.0) | 0.0 (0.0) | 0.598 | 0.805 |
| *[Eubacterium]_coprostanoligenes_group* | 0.7 (0.7) | 0.7 (0.4) | 0.630 | 0.823 |
| *[Eubacterium]_eligens_group* | 0.0 (0.2) | 0.1 (0.2) | 0.489 | 0.803 |
| *[Eubacterium]_hallii_group* | 1.4 (0.7) | 1.4 (0.6) | 0.359 | 0.685 |
| *[Eubacterium]_oxidoreducens_group* | 0.1 (0.0) | 0.1 (0.0) | 0.380 | 0.700 |
| *[Eubacterium]_ruminantium_group* | 0.0 (0.0) | 0.0 (0.0) | 0.317 | 0.670 |
| *[Eubacterium]_siraeum_group* | 0.0 (0.0) | 0.0 (0.1) | 0.248 | 0.670 |
| *[Eubacterium]_ventriosum_group* | 0.2 (0.1) | 0.2 (0.2) | 0.767 | 0.906 |
| *[Eubacterium]_xylanophilum_group* | 0.0 (0.2) | 0.0 (0.0) | 0.102 | 0.670 |
| *[Ruminococcus]_gauvreauii_group* | 0.2 (0.5) | 0.3 (0.6) | 0.312 | 0.670 |
| *[Ruminococcus]_gnavus_group* | 0.0 (0.0) | 0.0 (0.0) | 0.081 | 0.670 |
| *[Ruminococcus]_torques_group* | 1.0 (1.0) | 1.4 (1.5) | 0.198 | 0.670 |
| *Acidaminococcus* | 0.0 (0.0) | 0.0 (0.0) | 1.000 | 1.000 |
| *Actinomyces* | 0.0 (0.0) | 0.0 (0.0) | 0.958 | 1.000 |
| *Adlercreutzia* | 0.2 (0.3) | 0.2 (0.4) | 0.694 | 0.853 |
| *Agathobacter* | 3.6 (1.9) | 5.4 (4.7) | 0.172 | 0.670 |
| *Akkermansia* | 0.0 (0.5) | 0.0 (0.6) | 0.967 | 1.000 |
| *Alistipes* | 0.7 (0.8) | 0.7 (0.7) | 0.732 | 0.887 |
| *Allisonella* | 0.0 (0.1) | 0.0 (0.0) | 0.207 | 0.670 |
| *Alloprevotella* | 0.0 (0.0) | 0.0 (0.0) | 1.000 | 1.000 |
| *Anaerostipes* | 1.2 (0.7) | 1.6 (1.2) | 0.479 | 0.803 |
| *Anaerotruncus* | 0.0 (0.0) | 0.0 (0.0) | 0.571 | 0.803 |
| *Bacteroides* | 6.0 (4.7) | 4.6 (3.6) | 0.301 | 0.670 |
| *Barnesiella* | 0.5 (1.1) | 0.4 (0.6) | 0.178 | 0.670 |
| *Bifidobacterium* | 4.6 (3.4) | 6.0 (7.7) | 0.338 | 0.670 |
| *Bilophila* | 0.1 (0.2) | 0.1 (0.1) | 0.925 | 1.000 |
| *Blautia* | 5.3 (2.6) | 5.8 (1.7) | 0.243 | 0.670 |
| *Brachyspira* | 0.0 (0.0) | 0.0 (0.0) | 0.334 | 0.670 |
| *Butyricicoccus* | 0.4 (0.4) | 0.5 (0.3) | 0.097 | 0.670 |
| *Butyricimonas* | 0.2 (0.1) | 0.2 (0.1) | 0.936 | 1.000 |
| *Butyrivibrio* | 0.0 (0.0) | 0.0 (0.0) | 0.543 | 0.803 |
| *CAG-352* | 1.5 (2.7) | 0.3 (1.4) | 0.172 | 0.670 |
| *CAG-56* | 0.3 (0.5) | 0.4 (0.5) | 0.319 | 0.670 |
| *Candidatus_Soleaferrea* | 0.0 (0.0) | 0.0 (0.0) | 0.334 | 0.670 |
| *Catenibacterium* | 0.1 (0.0) | 0.1 (1.5) | 0.382 | 0.700 |
| *CHKCI002* | 0.0 (0.0) | 0.0 (0.0) | 0.161 | 0.670 |
| *Christensenellaceae_R-7_group* | 0.7 (0.7) | 0.7 (1.1) | 0.988 | 1.000 |
| *Citrobacter* | 0.0 (0.0) | 0.0 (0.0) | 0.334 | 0.670 |
| *Clostridia_UCG-014* | 0.5 (0.8) | 0.6 (0.7) | 0.561 | 0.803 |
| *Clostridia_vadinBB60_group* | 0.0 (0.0) | 0.0 (0.0) | 0.334 | 0.670 |
| *Clostridium_sensu_stricto_1* | 0.9 (1.2) | 0.5 (0.7) | 0.023 | 0.670 |
| *Colidextribacter* | 0.1 (0.2) | 0.1 (0.1) | 0.873 | 1.000 |
| *Collinsella* | 5.8 (2.9) | 7.0 (4.7) | 0.330 | 0.670 |
| *Coprobacillus* | 0.0 (0.0) | 0.0 (0.0) | 0.161 | 0.670 |
| *Coprobacter* | 0.0 (0.0) | 0.0 (0.0) | 0.986 | 1.000 |
| *Coprococcus* | 2.3 (1.4) | 2.3 (1.2) | 0.237 | 0.670 |
| *Defluviitaleaceae_UCG-011* | 0.0 (0.0) | 0.0 (0.0) | 0.334 | 0.670 |
| *Desulfovibrio* | 0.3 (0.5) | 0.0 (0.4) | 0.194 | 0.670 |
| *Dialister* | 2.7 (4.1) | 2.0 (4.3) | 0.601 | 0.805 |
| *Dielma* | 0.0 (0.0) | 0.0 (0.0) | 1.000 | 1.000 |
| *Dorea* | 1.8 (0.8) | 1.9 (1.0) | 0.335 | 0.670 |
| *DTU014* | 0.0 (0.0) | 0.0 (0.0) | 0.334 | 0.670 |
| *DTU089* | 0.0 (0.0) | 0.0 (0.0) | 0.288 | 0.670 |
| *Eggerthella* | 0.0 (0.0) | 0.0 (0.0) | 0.305 | 0.670 |
| *Enorma* | 0.0 (0.0) | 0.0 (0.0) | 0.334 | 0.670 |
| *Enterobacter* | 0.0 (0.0) | 0.0 (0.0) | 0.334 | 0.670 |
| *Enterococcus* | 0.0 (0.0) | 0.0 (0.0) | 1.000 | 1.000 |
| *Enterorhabdus* | 0.0 (0.0) | 0.0 (0.1) | 0.548 | 0.803 |
| *Erysipelatoclostridium* | 0.1 (0.1) | 0.1 (0.1) | 0.866 | 1.000 |
| *Erysipelotrichaceae_UCG-003* | 1.0 (1.3) | 0.8 (1.1) | 0.726 | 0.886 |
| *Escherichia-Shigella* | 0.0 (0.0) | 0.0 (0.0) | 0.694 | 0.853 |
| *Ezakiella* | 0.0 (0.0) | 0.0 (0.0) | 0.334 | 0.670 |
| *Faecalibacterium* | 9.2 (5.0) | 8.7 (3.2) | 0.315 | 0.670 |
| *Faecalitalea* | 0.0 (0.0) | 0.0 (0.0) | 0.305 | 0.670 |
| *Family_XIII_AD3011_group* | 0.1 (0.1) | 0.0 (0.1) | 0.384 | 0.700 |
| *Family_XIII_UCG-001* | 0.0 (0.1) | 0.0 (0.1) | 0.901 | 1.000 |
| *Flavonifractor* | 0.0 (0.0) | 0.0 (0.0) | 0.678 | 0.853 |
| *Fournierella* | 0.0 (0.0) | 0.0 (0.0) | 1.000 | 1.000 |
| *Frisingicoccus* | 0.0 (0.0) | 0.0 (0.0) | 1.000 | 1.000 |
| *Fructilactobacillus* | 0.0 (0.0) | 0.0 (0.0) | 0.334 | 0.670 |
| *Fusicatenibacter* | 2.0 (1.4) | 1.4 (1.3) | 0.300 | 0.670 |
| *Gastranaerophilales* | 0.0 (0.0) | 0.0 (0.0) | 0.598 | 0.805 |
| *GCA-900066575* | 0.0 (0.0) | 0.0 (0.0) | 0.571 | 0.803 |
| *Gordonibacter* | 0.0 (0.0) | 0.0 (0.0) | 0.288 | 0.670 |
| *Haemophilus* | 0.0 (0.1) | 0.0 (0.0) | 0.010 | 0.670 |
| *Holdemanella* | 1.1 (3.6) | 2.4 (3.7) | 0.524 | 0.803 |
| *Holdemania* | 0.0 (0.0) | 0.0 (0.0) | 1.000 | 1.000 |
| *Howardella* | 0.0 (0.1) | 0.0 (0.0) | 0.043 | 0.670 |
| *Incertae_Sedis* | 0.4 (0.5) | 0.3 (0.2) | 0.118 | 0.670 |
| *Intestinibacter* | 0.3 (0.7) | 0.0 (0.4) | 0.076 | 0.670 |
| *Intestinimonas* | 0.0 (0.0) | 0.0 (0.0) | 0.334 | 0.670 |
| *Izemoplasmatales* | 0.0 (0.0) | 0.0 (0.0) | 0.543 | 0.803 |
| *Lachnoclostridium* | 0.4 (0.4) | 0.7 (0.6) | 0.176 | 0.670 |
| *Lachnospira* | 0.2 (0.3) | 0.2 (0.3) | 0.174 | 0.670 |
| *Lachnospiraceae_FCS020_group* | 0.3 (0.2) | 0.2 (0.3) | 0.045 | 0.670 |
| *Lachnospiraceae_NC2004_group* | 0.1 (0.0) | 0.1 (0.0) | 0.628 | 0.823 |
| *Lachnospiraceae_ND3007_group* | 0.6 (0.4) | 0.4 (0.4) | 0.096 | 0.670 |
| *Lachnospiraceae_NK4A136_group* | 0.6 (0.7) | 0.5 (0.8) | 0.163 | 0.670 |
| *Lachnospiraceae_UCG-001* | 0.0 (0.2) | 0.0 (0.2) | 0.357 | 0.685 |
| *Lachnospiraceae_UCG-004* | 0.1 (0.0) | 0.1 (0.0) | 0.770 | 0.906 |
| *Lachnospiraceae_UCG-008* | 0.0 (0.0) | 0.0 (0.0) | 0.517 | 0.803 |
| *Lachnospiraceae_UCG-010* | 0.0 (0.1) | 0.0 (0.1) | 0.492 | 0.803 |
| *Lacticaseibacillus* | 0.0 (0.0) | 0.0 (0.0) | 0.339 | 0.670 |
| *Lactobacillus* | 0.0 (0.0) | 0.0 (0.0) | 1.000 | 1.000 |
| *Lactococcus* | 0.0 (0.0) | 0.0 (0.0) | 0.042 | 0.670 |
| *Latilactobacillus* | 0.0 (0.0) | 0.0 (0.0) | 0.598 | 0.805 |
| *Libanicoccus* | 0.0 (0.0) | 0.0 (0.0) | 0.742 | 0.892 |
| *Ligilactobacillus* | 0.0 (0.0) | 0.0 (0.0) | 0.370 | 0.697 |
| *Limosilactobacillus* | 0.0 (0.0) | 0.0 (0.0) | 0.571 | 0.803 |
| *Mailhella* | 0.0 (0.0) | 0.0 (0.0) | 0.334 | 0.670 |
| *Marvinbryantia* | 0.0 (0.0) | 0.0 (0.0) | 0.639 | 0.829 |
| *Megamonas* | 0.0 (0.0) | 0.0 (0.0) | 0.613 | 0.815 |
| *Megasphaera* | 0.0 (0.0) | 0.0 (0.0) | 0.296 | 0.670 |
| *Merdibacter* | 0.0 (0.0) | 0.0 (0.0) | 0.958 | 1.000 |
| *Methanobrevibacter* | 0.0 (0.2) | 0.0 (0.6) | 0.673 | 0.853 |
| *Methanosphaera* | 0.0 (0.0) | 0.0 (0.0) | 1.000 | 1.000 |
| *Mitsuokella* | 0.0 (0.0) | 0.0 (0.0) | 1.000 | 1.000 |
| *Mogibacterium* | 0.0 (0.0) | 0.0 (0.0) | 0.334 | 0.670 |
| *Monoglobus* | 0.6 (0.5) | 0.3 (0.4) | 0.017 | 0.670 |
| *Moryella* | 0.0 (0.0) | 0.0 (0.0) | 0.543 | 0.803 |
| *Muribaculaceae* | 0.0 (0.1) | 0.0 (0.0) | 0.088 | 0.670 |
| *Negativibacillus* | 0.0 (0.0) | 0.0 (0.0) | 0.675 | 0.853 |
| *NK4A214_group* | 0.3 (0.3) | 0.1 (0.2) | 0.051 | 0.670 |
| *Odoribacter* | 0.2 (0.2) | 0.1 (0.2) | 0.820 | 0.959 |
| *Olsenella* | 0.0 (0.1) | 0.0 (0.0) | 1.000 | 1.000 |
| *Oscillibacter* | 0.0 (0.1) | 0.0 (0.1) | 0.993 | 1.000 |
| *Oxalobacter* | 0.0 (0.0) | 0.0 (0.0) | 0.161 | 0.670 |
| *Parabacteroides* | 0.9 (0.5) | 0.8 (0.5) | 0.293 | 0.670 |
| *Paraprevotella* | 0.4 (0.5) | 0.1 (0.3) | 0.198 | 0.670 |
| *Parasutterella* | 0.0 (0.0) | 0.0 (0.0) | 0.597 | 0.805 |
| *Parvibacter* | 0.0 (0.0) | 0.0 (0.0) | 0.543 | 0.803 |
| *Peptococcus* | 0.0 (0.0) | 0.0 (0.0) | 0.655 | 0.843 |
| *Peptostreptococcaceae* | 0.0 (0.0) | 0.0 (0.0) | 0.334 | 0.670 |
| *Phascolarctobacterium* | 0.1 (1.0) | 0.7 (1.8) | 0.045 | 0.670 |
| *Phocea* | 0.0 (0.0) | 0.0 (0.0) | 0.334 | 0.670 |
| *Phoenicibacter* | 0.0 (0.0) | 0.0 (0.0) | 0.334 | 0.670 |
| *Porphyromonas* | 0.0 (0.0) | 0.0 (0.0) | 0.161 | 0.670 |
| *Prevotella* | 0.0 (0.0) | 0.0 (0.0) | 0.543 | 0.803 |
| *Prevotella_7* | 0.1 (1.0) | 0.1 (1.2) | 0.694 | 0.853 |
| *Prevotella_9* | 0.5 (5.4) | 0.7 (8.9) | 0.504 | 0.803 |
| *Prevotellaceae_Ga6A1_group* | 0.0 (0.0) | 0.0 (0.0) | 0.334 | 0.670 |
| *Prevotellaceae_NK3B31_group* | 0.0 (0.0) | 0.0 (0.0) | 0.402 | 0.714 |
| *RF39* | 0.1 (0.1) | 0.1 (0.1) | 0.470 | 0.803 |
| *Rikenellaceae_RC9_gut_group* | 0.0 (0.0) | 0.0 (0.0) | 0.416 | 0.728 |
| *Romboutsia* | 1.3 (1.3) | 1.0 (1.4) | 0.162 | 0.670 |
| *Roseburia* | 1.2 (2.1) | 0.5 (1.5) | 0.075 | 0.670 |
| *Rothia* | 0.0 (0.0) | 0.0 (0.0) | 0.334 | 0.670 |
| *Ruminococcus* | 1.4 (1.0) | 1.1 (1.3) | 0.359 | 0.685 |
| *Sanguibacteroides* | 0.0 (0.0) | 0.0 (0.0) | 0.334 | 0.670 |
| *Sarcina* | 0.0 (0.0) | 0.0 (0.0) | 0.571 | 0.803 |
| *Sellimonas* | 0.0 (0.0) | 0.0 (0.0) | 0.334 | 0.670 |
| *Senegalimassilia* | 0.0 (0.4) | 0.0 (0.4) | 0.751 | 0.897 |
| *Slackia* | 0.2 (0.4) | 0.2 (0.3) | 1.000 | 1.000 |
| *Solobacterium* | 0.0 (0.0) | 0.0 (0.0) | 1.000 | 1.000 |
| *Staphylococcus* | 0.0 (0.0) | 0.0 (0.0) | 0.334 | 0.670 |
| *Streptococcus* | 0.6 (1.1) | 0.5 (0.6) | 0.392 | 0.708 |
| *Subdoligranulum* | 3.1 (1.3) | 2.6 (2.0) | 0.258 | 0.670 |
| *Succinivibrio* | 0.0 (0.0) | 0.0 (0.0) | 0.334 | 0.670 |
| *Sutterella* | 0.2 (0.2) | 0.3 (0.5) | 0.535 | 0.803 |
| *Terrisporobacter* | 0.0 (0.3) | 0.0 (0.0) | 0.029 | 0.670 |
| *Turicibacter* | 0.0 (0.2) | 0.0 (0.1) | 0.463 | 0.801 |
| *Tyzzerella* | 0.0 (0.0) | 0.0 (0.0) | 0.111 | 0.670 |
| *UBA1819* | 0.0 (0.0) | 0.0 (0.0) | 0.288 | 0.670 |
| *UCG-002* | 0.6 (0.7) | 0.6 (0.5) | 0.205 | 0.670 |
| *UCG-003* | 0.0 (0.1) | 0.0 (0.0) | 0.167 | 0.670 |
| *UCG-005* | 0.1 (0.5) | 0.0 (0.1) | 0.405 | 0.714 |
| *UCG-008* | 0.0 (0.0) | 0.0 (0.0) | 0.161 | 0.670 |
| *UCG-009* | 0.0 (0.0) | 0.0 (0.0) | 0.161 | 0.670 |
| *UCG-010* | 0.1 (0.0) | 0.1 (0.0) | 0.540 | 0.803 |
| uncultured | 1.3 (0.6) | 1.2 (0.6) | 0.555 | 0.803 |
| *vadinBE97* | 0.0 (0.0) | 0.0 (0.0) | 0.334 | 0.670 |
| *Veillonella* | 0.0 (0.0) | 0.0 (0.0) | 0.598 | 0.805 |
| *Victivallis* | 0.0 (0.0) | 0.0 (0.0) | 0.161 | 0.670 |
| *Weissella* | 0.0 (0.0) | 0.0 (0.0) | 0.334 | 0.670 |

Between group differences are assessed by Mann-Whitney U and corrected for multiple comparisons using the Benjamini and Hochberg false discovery rate correction (*p*_adj_ value).

**Supplementary Table 6.** Biomarkers of gastrointestinal barrier integrity, and inflammation at baseline and post-heat tolerance assessment for control participants without previous Exertional Heat Illness (EHI) history (Control: *n*=29) and recent EHI patients (Patient: *n*=29).

|  | **Control** | | **Patient** | | ***p* value; Effect size** | | |
| --- | --- | --- | --- | --- | --- | --- | --- |
|  | Baseline | Post-HTA | Baseline | Post-HTA^a^ | Timepoint | Group | Interaction |
| I-FABP _Log_ (pg·mL^-1^) | 6.33 ± 0.19 | 7.07 ± 0.19 | 6.23 ± 0.19 | 6.94 ± 0.19 | <0.001***; 0.60 | 0.647; <0.01 | 0.874; <0.01 |
| *I-FABP (pg·mL^-1^)* | *402.04 (568.89)* | *1238.89 (1246.54)* | *421.18 (713.85)* | *944.45 (2067.80)* | - | - | - |
| CLDN3 (ng·mL^-1^) | 8.11 ± 0.36 | 6.99 ± 0.36 | 7.36 ± 0.36 | 6.44 ± 0.37 | <0.001***; 0.37 | 0.173; 0.03 | 0.572; 0.01 |
| LBP _Log_ (µg·mL^-1^) | 1.12 ± 0.11 | 1.11 ± 0.11 | 0.95 ± 0.11 | 0.96 ± 0.11 | 0.765; <0.01 | 0.322; 0.02 | 0.394; 0.01 |
| *LBP (µg·mL^-1^)* | *3.44 ± 1.58* | *3.41 ± 1.58* | *2.97 ± 1.30* | *3.05 ± 1.30* | - | - | - |
| sCD14 (µg·mL^-1^) | 1.44 ± 0.06 | 1.47 ± 0.06 | 1.32 ± 0.06 | 1.34 ± 0.06 | 0.393; 0.01 | 0.127; 0.04 | 0.699; <0.01 |
| IL-6 _Log_ (pg·mL^-1^) | -0.51 ± 0.12 | 1.21 ± 0.12 | -0.57 ± 0.12 | 1.32 ± 0.12 | <0.001***; 0.86 | 0.859; <0.01 | 0.377; 0.01 |
| *IL-6 (pg·mL^-1^)* | *0.56 (0.72)* | *3.13 (1.83)* | *0.48 (0.52)* | *3.85 (2.26)* | - | - | - |

Data are presented as estimated marginal means ± SE. Effect size is presented as partial eta squared (*η*^2^*_p_*). Where data has been log transformed, the raw data is presented as mean ± SD for parametric data, or median (IQR) for non-parametric data. I-FABP=intestinal fatty acid binding protein; CLDN-3=claudin 3; LBP=liposaccharide binding protein; sCD14=soluble Cluster of Differentiation 14; IL-6=interleukin 6. ^a^ *n*=28; ***=*p*<0.001.

**Supplementary Table 7.** Thermophysiological responses during the Heat Tolerance Assessment for heat tolerant (*n*=46) and heat intolerant (*n*=11) individuals.

| **Variable** | **Heat tolerant** | **Heat intolerant** | ***p* value** | **Effect size** |
| --- | --- | --- | --- | --- |
| **Rectal temperature** |  |  |  |  |
| Phase 1 (°C^.^h^-1^) | 1.90 (0.40) | 1.90 (0.65) | 0.320 | *r* = 0.20 |
| Phase 2 (°C^.^h^-1^) | 0.70 (0.35) | 1.20 (0.83) | <0.001*** | *r* = 0.80 |
| Termination (°C) | 38.80 ± 0.31 | 39.20 ± 0.31 | <0.001*** | *d* = -1.32 |
| **Mean skin temperature** |  |  |  |  |
| Phase 1 (°C·h^-1^) | 3.87 (2.60)^b^ | 5.64 (3.76) ^d^ | 0.176 | *r* = 0.31 |
| Phase 2 (°C·h^-1^) | -3.00 (2.10)^b^ | -1.13 (1.87) ^d^ | 0.223 | *r* = 0.28 |
| Termination (°C) | 34.07 ± 1.00^b^ | 34.48 ± 0.98 ^d^ | 0.293 | *d* = -0.41 |
| **Mean body temperature** |  |  |  |  |
| Phase 1 (°C^.^h^-1^) | 2.07 (0.40)^b^ | 2.19 (0.79) ^d^ | 0.368 | *r* = 0.21 |
| Phase 2 (°C^.^h^-1^) | 0.32 ± 0.28^b^ | 0.87 ± 0.32 ^d^ | <0.001*** | *d* = -1.89 |
| Termination (°C) | 38.34 ± 0.31^b^ | 38.74 ± 0.28 ^d^ | 0.001** | *d* = -1.32 |
| **Heart rate** |  |  |  |  |
| Phase 1 (beats·min^-1^·h^-1^) | 145 (26) | 142 (44) | 0.824 | *r* = 0.05 |
| Phase 2 (beats·min^-1^·h^-1^) | -28 (25)^a^ | -5 (21)^c^ | 0.025* | *r* = 0.44 |
| Termination (beats^.^min^-1^) | 157 (17)^a^ | 173 (18)^c^ | 0.020* | *r* = 0.46 |
| **Sweat rate** |  |  |  |  |
| WBSR (L·m^-2^·h^-1^) | 0.62 (0.20) | 0.53 (0.17) | 0.011* | *r* = 0.49 |

Normally distributed data are presented as mean ± SD with between groups difference and effect size assessed by independent samples t-test and Cohen’s d, respectively. Non-normally distributed data are presented as median (IQR) with between groups difference and effect size assessed by Mann-Whitney U test and rank biserial correlation, respectively. WBSR=whole body sweat rate. ^a^ *n*=45; ^b^ *n*=42; ^c^ *n*=11; ^d^ *n*=8. *=*p*<0.050; **=*p*<0.010; ***=*p*<0.001.

**Supplementary Table 8.** Perceptual variables during the heat tolerance assessment for heat tolerant *(n*=46) and heat intolerant (*n*=12) individuals.

| **Variable** | **Heat tolerant** | **Heat intolerant** | ***p* value** | **Effect size** |
| --- | --- | --- | --- | --- |
| **RPE (scale: 6 – 20)** |  |  |  |  |
| Δ Phase 1 | 6.9 ± 2.1 | 7.6 ± 2.3 | 0.300 | *d* = -0.35 |
| Δ Phase 2 | -3.7 ± 2.0 | 0.5 ± 2.3 | <0.001*** | *d* = -2.01 |
| Termination | 10.0 (2.8) | 15.0 (2.0) | <0.001*** | *r* = 0.75 |
| **TC (scale 1.0 – 5.0)** |  |  |  |  |
| Δ Phase 1 | 1.5 (1.0) | 1.5 (0.8) | 0.238 | *r* = 0.23 |
| Δ Phase 2 | -1.0 (0.5) | 0.5 (0.5) | <0.001*** | *r* = 0.75 |
| Termination | 2.0 (0.9) | 4.0 (0.8) | <0.001*** | *r* = 0.85 |
| **TS (scale 5 – 13)** |  |  |  |  |
| Δ Phase 1 | 2.0 (1.8) | 3.0 (2.0) | 0.015* | *r* = 0.45 |
| Δ Phase 2 | -1.5 (1.0) | 0.0 (2.0) | 0.001** | *r* = 0.62 |
| Termination | 8.0 (1.0) | 11.0 (2.0) | <0.001*** | *r* = 0.75 |

Normally distributed data are presented as mean ± SD with between groups difference and effect size assessed by independent samples t-test and Cohen’s d, respectively. Non-normally distributed data are presented as median (IQR) with between groups difference and effect size assessed by Mann-Whitney U test and rank biserial correlation, respectively. RPE=Rating of perceived exertion; TC= Thermal comfort; TS=Thermal sensation. *=*p*<0.050; **=*p*<0.010; ***=*p*<0.001.

**Supplementary Table 9.** Median (IQR) gastrointestinal symptoms pre- and post-Heat Tolerance Assessment for heat tolerant (*n*=46) and heat intolerant *(n*=12) individuals.

| **Variable** | **Heat tolerant** | **Heat intolerant** | ***p* value** | **Effect size** |
| --- | --- | --- | --- | --- |
| **Pre-HTA GIS** |  |  |  |  |
| Gut discomfort | 0.0 (0.0) | 0.0 (0.0) | 0.565 | *r* = 0.04 |
| Overall GIS | 0.0 (0.0) | 0.0 (1.0) | 0.461 | *r* = 0.11 |
| Upper GIS | 0.0 (0.0) | 0.0 (0.0) | 0.302 | *r* = 0.06 |
| Lower GIS | 0.0 (0.0) | 0.0 (0.0) | 0.279 | *r* = 0.14 |
| Nausea | 0.0 (0.0) | 0.0 (0.0) | 0.055 | *r* = 0.08 |
| Dizziness | 0.0 (0.0) | 0.0 (0.3) | 0.001** | *r* = 0.25 |
| **Post-HTA GIS ^a^** |  |  |  |  |
| Gut discomfort | 0.0 (0.0) | 0.0 (1.0)^a^ | 0.142 | *r* = 0.20 |
| Overall GIS | 0.5 (3.0) | 8.0 (8.0)^a^ | <0.001*** | *r* = 0.67 |
| Upper GIS | 0.0 (0.0) | 0.0 (1.0)^a^ | 0.496 | *r* = 0.10 |
| Lower GIS | 0.0 (1.0) | 0.0 (1.0)^a^ | 0.866 | *r* = 0.03 |
| Nausea | 0.0 (0.0) | 3.0 (4.0)^a^ | <0.001*** | *r* = 0.77 |
| Dizziness | 0.0 (1.0) | 4.0 (5.5)^a^ | <0.001*** | *r* = 0.75 |

Between groups difference and effect size assessed by Mann-Whitney U test and rank biserial correlation, respectively. GIS=gastrointestinal symptoms; HTA=heat tolerance assessment.  ^a^ *n*=11. **=*p*<0.010; ***=*p*<0.001.

**Supplementary Table 10.** Number of bacterial taxa detected at each taxonomic level for individuals classified as heat tolerant (*n*=46) or heat intolerant (*n*=12).

| **Group** | **Phyla** | **Classes** | **Orders** | **Families** | **Genera** | **ASVs** |
| --- | --- | --- | --- | --- | --- | --- |
| **Heat tolerant** | 6 (1) | 9 (2) | 17 ± 2 | 29 ± 4 | 60 ± 8 | 152 ± 32 |
| **Heat intolerant** | 6 (1) | 10 (1) | 17 ± 2 | 28 ± 4 | 61 ± 7 | 160 ± 33 |

Normally distributed data are presented as mean ± SD. Non-normally distributed data are presented as median (IQR). ASV, amplicon sequence variant.

**Supplementary Table 11.** Median (IQR) relative abundance of bacterial taxa at a Phylum, Class, Order, Family, and Genus taxonomic level for individuals classified as heat tolerant (*n*=46) or heat intolerant (*n*=12).

| **Taxa** | **Tolerant** | **Intolerant** | ***p* value** | ***p*_adj_ value** |
| --- | --- | --- | --- | --- |
| **Phylum** |  |  |  |  |
| Actinobacteriota | 13.3 (8.8) | 15.2 (6.3) | 0.442 | 0.640 |
| Bacteroidota | 13.8 (6.2) | 16.7 (3.4) | 0.046 | 0.312 |
| Cyanobacteria | 0.0 (0.0) | 0.0 (0.0) | 0.381 | 0.640 |
| Desulfobacterota | 0.4 (0.4) | 0.3 (0.3) | 0.578 | 0.640 |
| Euryarchaeota | 0.0 (0.3) | 0.0 (1.0) | 0.245 | 0.640 |
| Firmicutes | 66.6 (8.2) | 61.8 (5.4) | 0.069 | 0.312 |
| Proteobacteria | 0.5 (0.5) | 0.5 (0.7) | 0.382 | 0.640 |
| Spirochaetota | 0.0 (0.0) | 0.0 (0.0) | 0.640 | 0.640 |
| Verrucomicrobiota | 0.1 (0.6) | 0.1 (0.6) | 0.580 | 0.640 |
| **Class** |  |  |  |  |
| Actinobacteria | 5.0 (6.6) | 4.7 (5.9) | 0.828 | 0.828 |
| Alphaproteobacteria | 0.0 (0.0) | 0.0 (0.0) | 0.481 | 0.685 |
| Bacilli | 6.2 (4.3) | 5.5 (4.7) | 0.502 | 0.685 |
| Bacteroidia | 13.8 (6.2) | 16.7 (3.4) | 0.046 | 0.227 |
| Brachyspirae | 0.0 (0.0) | 0.0 (0.0) | 0.640 | 0.685 |
| Clostridia | 52.9 (11.0) | 49.1 (9.3) | 0.061 | 0.227 |
| Coriobacteriia | 7.7 (3.6) | 9.1 (3.9) | 0.172 | 0.516 |
| Desulfovibrionia | 0.4 (0.4) | 0.3 (0.3) | 0.578 | 0.685 |
| Gammaproteobacteria | 0.5 (0.5) | 0.5 (0.7) | 0.278 | 0.595 |
| Incertae_Sedis | 0.0 (0.0) | 0.0 (0.0) | 0.640 | 0.685 |
| Lentisphaeria | 0.0 (0.0) | 0.0 (0.0) | 0.006 | 0.086 |
| Methanobacteria | 0.0 (0.3) | 0.0 (1.0) | 0.245 | 0.595 |
| Negativicutes | 4.0 (3.1) | 7.1 (6.9) | 0.058 | 0.227 |
| Vampirivibrionia | 0.0 (0.0) | 0.0 (0.0) | 0.381 | 0.685 |
| Verrucomicrobiae | 0.0 (0.6) | 0.1 (0.6) | 0.580 | 0.685 |
| **Order** |  |  |  |  |
| Acidaminococcales | 0.6 (1.3) | 0.6 (1.2) | 0.646 | 0.761 |
| Actinomycetales | 0.0 (0.0) | 0.0 (0.0) | 0.305 | 0.745 |
| Bacteroidales | 13.8 (6.2) | 16.6 (3.4) | 0.048 | 0.261 |
| Bifidobacteriales | 5.0 (6.6) | 4.7 (5.9) | 0.828 | 0.881 |
| Brachyspirales | 0.0 (0.0) | 0.0 (0.0) | 0.640 | 0.761 |
| Burkholderiales | 0.3 (0.3) | 0.4 (0.7) | 0.269 | 0.739 |
| Christensenellales | 0.8 (0.8) | 0.6 (1.0) | 0.437 | 0.761 |
| Clostridia_UCG-014 | 0.6 (0.7) | 0.2 (0.8) | 0.211 | 0.696 |
| Clostridia_vadinBB60_group | 0.0 (0.0) | 0.0 (0.0) | 0.640 | 0.761 |
| Clostridiales | 0.6 (1.2) | 0.8 (1.5) | 0.339 | 0.745 |
| Coriobacteriales | 7.7 (3.6) | 9.1 (3.9) | 0.172 | 0.631 |
| Desulfovibrionales | 0.4 (0.4) | 0.3 (0.3) | 0.578 | 0.761 |
| DTU014 | 0.0 (0.0) | 0.0 (0.0) | 0.640 | 0.761 |
| Enterobacterales | 0.1 (0.2) | 0.1 (0.2) | 0.819 | 0.881 |
| Erysipelotrichales | 4.5 (4.8) | 5.0 (4.9) | 0.917 | 0.917 |
| Flavobacteriales | 0.0 (0.0) | 0.0 (0.0) | 0.055 | 0.261 |
| Gastranaerophilales | 0.0 (0.0) | 0.0 (0.0) | 0.381 | 0.761 |
| Izemoplasmatales | 0.0 (0.0) | 0.0 (0.0) | 0.599 | 0.761 |
| Lachnospirales | 28.4 (6.4) | 26.9 (4.9) | 0.082 | 0.340 |
| Lactobacillales | 1.0 (1.6) | 0.4 (0.3) | 0.004 | 0.095 |
| Methanobacteriales | 0.0 (0.3) | 0.0 (1.0) | 0.245 | 0.735 |
| Micrococcales | 0.0 (0.0) | 0.0 (0.0) | 0.640 | 0.761 |
| Monoglobales | 0.5 (0.4) | 0.4 (0.5) | 0.477 | 0.761 |
| Opitutales | 0.0 (0.0) | 0.0 (0.0) | 0.055 | 0.261 |
| Oscillospirales | 20.4 (5.8) | 19.7 (5.9) | 0.328 | 0.745 |
| Peptococcales | 0.0 (0.0) | 0.0 (0.0) | 0.623 | 0.761 |
| Peptostreptococcales-Tissierellales | 1.7 (2.3) | 2.1 (1.4) | 0.513 | 0.761 |
| RF39 | 0.1 (0.1) | 0.1 (0.1) | 0.783 | 0.881 |
| Rhodospirillales | 0.0 (0.0) | 0.0 (0.0) | 0.481 | 0.761 |
| Staphylococcales | 0.0 (0.0) | 0.0 (0.0) | 0.055 | 0.261 |
| Veillonellales-Selenomonadales | 2.6 (4.3) | 6.6 (5.8) | 0.044 | 0.261 |
| Verrucomicrobiales | 0.0 (0.6) | 0.1 (0.6) | 0.893 | 0.917 |
| Victivallales | 0.0 (0.0) | 0.0 (0.0) | 0.006 | 0.095 |
| **Family** |  |  |  |  |
| *[Clostridium]_methylpentosum_group* | 0.0 (0.0) | 0.0 (0.0) | 0.640 | 0.731 |
| *[Eubacterium]_coprostanoligenes_group* | 0.7 (0.5) | 0.6 (1.2) | 0.584 | 0.731 |
| *Acidaminococcaceae* | 0.6 (1.3) | 0.6 (1.2) | 0.646 | 0.731 |
| *Actinomycetaceae* | 0.0 (0.0) | 0.0 (0.0) | 0.305 | 0.731 |
| *Akkermansiaceae* | 0.0 (0.6) | 0.1 (0.6) | 0.893 | 0.908 |
| *Anaerovoracaceae* | 0.1 (0.1) | 0.1 (0.2) | 0.809 | 0.867 |
| *Atopobiaceae* | 0.1 (0.4) | 0.1 (0.0) | 0.313 | 0.731 |
| *Bacteroidaceae* | 4.6 (4.9) | 5.6 (3.9) | 0.840 | 0.869 |
| *Barnesiellaceae* | 0.5 (0.6) | 0.9 (0.9) | 0.189 | 0.725 |
| *Bifidobacteriaceae* | 5.0 (6.6) | 4.7 (5.9) | 0.828 | 0.869 |
| *Brachyspiraceae* | 0.0 (0.0) | 0.0 (0.0) | 0.640 | 0.731 |
| *Butyricicoccaceae* | 0.5 (0.4) | 0.5 (0.4) | 0.513 | 0.731 |
| *Christensenellaceae* | 0.8 (0.8) | 0.6 (1.0) | 0.437 | 0.731 |
| *Clostridia_UCG-014* | 0.6 (0.7) | 0.2 (0.8) | 0.211 | 0.725 |
| *Clostridia_vadinBB60_group* | 0.0 (0.0) | 0.0 (0.0) | 0.640 | 0.731 |
| *Clostridiaceae* | 0.6 (1.2) | 0.8 (1.5) | 0.339 | 0.731 |
| *Coriobacteriaceae* | 6.0 (3.4) | 8.0 (4.7) | 0.072 | 0.434 |
| *Coriobacteriales_Incertae_Sedis* | 0.0 (0.1) | 0.0 (0.0) | 0.055 | 0.370 |
| *Defluviitaleaceae* | 0.0 (0.0) | 0.0 (0.0) | 0.640 | 0.731 |
| *Desulfovibrionaceae* | 0.4 (0.4) | 0.3 (0.3) | 0.578 | 0.731 |
| *DTU014* | 0.0 (0.0) | 0.0 (0.0) | 0.640 | 0.731 |
| *Eggerthellaceae* | 1.3 (0.9) | 1.1 (0.4) | 0.249 | 0.725 |
| *Enterobacteriaceae* | 0.0 (0.0) | 0.0 (0.1) | 0.402 | 0.731 |
| *Enterococcaceae* | 0.0 (0.0) | 0.0 (0.0) | 0.485 | 0.731 |
| *Erysipelatoclostridiaceae* | 1.9 (2.3) | 0.7 (0.9) | 0.005 | 0.172 |
| *Erysipelotrichaceae* | 2.0 (3.2) | 3.5 (2.5) | 0.278 | 0.725 |
| *Family_XI* | 0.0 (0.0) | 0.0 (0.0) | 0.640 | 0.731 |
| *Flavobacteriaceae* | 0.0 (0.0) | 0.0 (0.0) | 0.055 | 0.370 |
| *Gastranaerophilales* | 0.0 (0.0) | 0.0 (0.0) | 0.381 | 0.731 |
| *Hungateiclostridiaceae* | 0.0 (0.0) | 0.0 (0.0) | 0.640 | 0.731 |
| *Izemoplasmatales* | 0.0 (0.0) | 0.0 (0.0) | 0.599 | 0.731 |
| *Lachnospiraceae* | 28.4 (6.4) | 26.9 (4.9) | 0.082 | 0.449 |
| *Lactobacillaceae* | 0.0 (0.1) | 0.0 (0.0) | 0.043 | 0.370 |
| *Marinifilaceae* | 0.4 (0.3) | 0.3 (0.3) | 0.803 | 0.867 |
| *Methanobacteriaceae* | 0.0 (0.3) | 0.0 (1.0) | 0.245 | 0.725 |
| *Micrococcaceae* | 0.0 (0.0) | 0.0 (0.0) | 0.640 | 0.731 |
| *Monoglobaceae* | 0.5 (0.4) | 0.4 (0.5) | 0.477 | 0.731 |
| *Muribaculaceae* | 0.0 (0.0) | 0.0 (0.0) | 0.966 | 0.966 |
| *Oscillospiraceae* | 1.6 (1.3) | 1.2 (1.2) | 0.624 | 0.731 |
| *Oxalobacteraceae* | 0.0 (0.0) | 0.0 (0.0) | 0.565 | 0.731 |
| *Pasteurellaceae* | 0.0 (0.1) | 0.0 (0.0) | 0.645 | 0.731 |
| *Peptococcaceae* | 0.0 (0.0) | 0.0 (0.0) | 0.623 | 0.731 |
| *Peptostreptococcaceae* | 1.6 (2.3) | 1.9 (1.3) | 0.571 | 0.731 |
| *Porphyromonadaceae* | 0.0 (0.0) | 0.0 (0.0) | 0.331 | 0.731 |
| *Prevotellaceae* | 3.0 (6.7) | 6.2 (6.0) | 0.219 | 0.725 |
| *Puniceicoccaceae* | 0.0 (0.0) | 0.0 (0.0) | 0.055 | 0.370 |
| *RF39* | 0.1 (0.1) | 0.1 (0.1) | 0.783 | 0.867 |
| *Rikenellaceae* | 1.1 (0.8) | 0.9 (0.8) | 0.257 | 0.725 |
| *Ruminococcaceae* | 16.9 (5.5) | 16.3 (5.4) | 0.155 | 0.725 |
| *Selenomonadaceae* | 0.0 (0.0) | 0.0 (0.8) | 0.277 | 0.725 |
| *Staphylococcaceae* | 0.0 (0.0) | 0.0 (0.0) | 0.055 | 0.370 |
| *Streptococcaceae* | 0.6 (1.1) | 0.4 (0.3) | 0.012 | 0.245 |
| *Succinivibrionaceae* | 0.0 (0.0) | 0.0 (0.0) | 0.485 | 0.731 |
| *Sutterellaceae* | 0.3 (0.3) | 0.4 (0.7) | 0.260 | 0.725 |
| *Tannerellaceae* | 0.8 (0.6) | 0.8 (0.7) | 0.576 | 0.731 |
| *UCG-010* | 0.1 (0.0) | 0.1 (0.1) | 0.361 | 0.731 |
| uncultured | 0.0 (0.1) | 0.0 (0.0) | 0.200 | 0.725 |
| *vadinBE97* | 0.0 (0.0) | 0.0 (0.0) | 0.055 | 0.370 |
| *Veillonellaceae* | 2.6 (4.1) | 3.9 (4.8) | 0.243 | 0.725 |
| *Victivallaceae* | 0.0 (0.0) | 0.0 (0.0) | 0.006 | 0.172 |
| **Genus** |  |  |  |  |
| *[Clostridium]_innocuum_group* | 0.0 (0.0) | 0.0 (0.0) | 0.043 | 0.614 |
| *[Clostridium]_methylpentosum_group* | 0.0 (0.0) | 0.0 (0.0) | 0.640 | 0.811 |
| *[Eubacterium]_brachy_group* | 0.0 (0.0) | 0.0 (0.0) | 0.565 | 0.811 |
| *[Eubacterium]_coprostanoligenes_group* | 0.7 (0.5) | 0.6 (1.2) | 0.584 | 0.811 |
| *[Eubacterium]_eligens_group* | 0.0 (0.2) | 0.3 (0.3) | 0.089 | 0.804 |
| *[Eubacterium]_hallii_group* | 1.5 (0.5) | 1.0 (0.5) | 0.002 | 0.138 |
| *[Eubacterium]_oxidoreducens_group* | 0.1 (0.0) | 0.1 (0.0) | 0.481 | 0.811 |
| *[Eubacterium]_ruminantium_group* | 0.0 (0.0) | 0.0 (0.0) | 0.811 | 0.922 |
| *[Eubacterium]_siraeum_group* | 0.0 (0.1) | 0.0 (0.0) | 0.688 | 0.845 |
| *[Eubacterium]_ventriosum_group* | 0.2 (0.2) | 0.3 (0.1) | 0.171 | 0.811 |
| *[Eubacterium]_xylanophilum_group* | 0.0 (0.1) | 0.0 (0.0) | 0.462 | 0.811 |
| *[Ruminococcus]_gauvreauii_group* | 0.3 (0.5) | 0.3 (0.4) | 0.744 | 0.884 |
| *[Ruminococcus]_gnavus_group* | 0.0 (0.0) | 0.0 (0.0) | 0.565 | 0.811 |
| *[Ruminococcus]_torques_group* | 1.3 (1.1) | 0.8 (0.7) | 0.257 | 0.811 |
| *Acidaminococcus* | 0.0 (0.0) | 0.0 (0.0) | 0.431 | 0.811 |
| *Actinomyces* | 0.0 (0.0) | 0.0 (0.0) | 0.305 | 0.811 |
| *Adlercreutzia* | 0.2 (0.4) | 0.2 (0.2) | 0.812 | 0.922 |
| *Agathobacter* | 4.0 (4.5) | 4.8 (3.4) | 0.857 | 0.926 |
| *Akkermansia* | 0.0 (0.6) | 0.1 (0.6) | 0.893 | 0.932 |
| *Alistipes* | 0.7 (0.8) | 0.6 (0.7) | 0.460 | 0.811 |
| *Allisonella* | 0.0 (0.0) | 0.0 (0.1) | 0.447 | 0.811 |
| *Alloprevotella* | 0.0 (0.0) | 0.0 (0.0) | 0.793 | 0.921 |
| *Anaerostipes* | 1.5 (0.9) | 0.7 (0.3) | 0.003 | 0.138 |
| *Anaerotruncus* | 0.0 (0.0) | 0.0 (0.0) | 0.381 | 0.811 |
| *Bacteroides* | 4.6 (4.9) | 5.6 (3.9) | 0.840 | 0.926 |
| *Barnesiella* | 0.4 (0.6) | 0.7 (0.9) | 0.539 | 0.811 |
| *Bifidobacterium* | 5.0 (6.6) | 4.7 (5.9) | 0.828 | 0.922 |
| *Bilophila* | 0.1 (0.1) | 0.2 (0.2) | 0.543 | 0.811 |
| *Blautia* | 5.6 (2.3) | 4.3 (1.7) | 0.048 | 0.614 |
| *Brachyspira* | 0.0 (0.0) | 0.0 (0.0) | 0.640 | 0.811 |
| *Butyricicoccus* | 0.5 (0.4) | 0.5 (0.4) | 0.590 | 0.811 |
| *Butyricimonas* | 0.2 (0.1) | 0.2 (0.2) | 0.658 | 0.821 |
| *Butyrivibrio* | 0.0 (0.0) | 0.0 (0.0) | 0.381 | 0.811 |
| *CAG-352* | 0.8 (2.4) | 1.2 (1.4) | 0.929 | 0.962 |
| *CAG-56* | 0.3 (0.4) | 0.4 (0.6) | 0.561 | 0.811 |
| *Candidatus_Soleaferrea* | 0.0 (0.0) | 0.0 (0.0) | 0.055 | 0.614 |
| *Catenibacterium* | 0.1 (0.0) | 0.1 (0.4) | 0.864 | 0.926 |
| *CHKCI002* | 0.0 (0.0) | 0.0 (0.0) | 0.485 | 0.811 |
| *Christensenellaceae_R-7_group* | 0.7 (0.8) | 0.4 (1.0) | 0.342 | 0.811 |
| *Citrobacter* | 0.0 (0.0) | 0.0 (0.0) | 0.640 | 0.811 |
| *Clostridia_UCG-014* | 0.6 (0.7) | 0.2 (0.8) | 0.211 | 0.811 |
| *Clostridia_vadinBB60_group* | 0.0 (0.0) | 0.0 (0.0) | 0.640 | 0.811 |
| *Clostridium_sensu_stricto_1* | 0.5 (1.2) | 0.8 (1.5) | 0.309 | 0.811 |
| *Colidextribacter* | 0.1 (0.2) | 0.1 (0.1) | 0.622 | 0.811 |
| *Collinsella* | 6.0 (3.4) | 8.0 (4.7) | 0.072 | 0.751 |
| *Coprobacillus* | 0.0 (0.0) | 0.0 (0.0) | 0.485 | 0.811 |
| *Coprobacter* | 0.0 (0.0) | 0.1 (0.1) | 0.088 | 0.804 |
| *Coprococcus* | 2.5 (1.4) | 1.9 (1.1) | 0.097 | 0.804 |
| *Defluviitaleaceae_UCG-011* | 0.0 (0.0) | 0.0 (0.0) | 0.640 | 0.811 |
| *Desulfovibrio* | 0.2 (0.4) | 0.0 (0.3) | 0.357 | 0.811 |
| *Dialister* | 2.4 (4.1) | 3.1 (4.0) | 0.500 | 0.811 |
| *Dielma* | 0.0 (0.0) | 0.0 (0.0) | 0.485 | 0.811 |
| *Dorea* | 1.9 (0.8) | 1.9 (1.2) | 0.415 | 0.811 |
| *DTU014* | 0.0 (0.0) | 0.0 (0.0) | 0.640 | 0.811 |
| *DTU089* | 0.0 (0.0) | 0.0 (0.0) | 0.156 | 0.811 |
| *Eggerthella* | 0.0 (0.0) | 0.0 (0.0) | 0.861 | 0.926 |
| *Enorma* | 0.0 (0.0) | 0.0 (0.0) | 0.640 | 0.811 |
| *Enterobacter* | 0.0 (0.0) | 0.0 (0.0) | 0.640 | 0.811 |
| *Enterococcus* | 0.0 (0.0) | 0.0 (0.0) | 0.485 | 0.811 |
| *Enterorhabdus* | 0.0 (0.0) | 0.0 (0.1) | 0.201 | 0.811 |
| *Erysipelatoclostridium* | 0.1 (0.1) | 0.1 (0.1) | 0.624 | 0.811 |
| *Erysipelotrichaceae_UCG-003* | 1.1 (1.2) | 0.4 (0.4) | 0.001 | 0.123 |
| *Escherichia-Shigella* | 0.0 (0.0) | 0.0 (0.1) | 0.432 | 0.811 |
| *Ezakiella* | 0.0 (0.0) | 0.0 (0.0) | 0.640 | 0.811 |
| *Faecalibacterium* | 9.1 (3.9) | 8.5 (2.2) | 0.465 | 0.811 |
| *Faecalitalea* | 0.0 (0.0) | 0.0 (0.0) | 0.827 | 0.922 |
| *Family_XIII_AD3011_group* | 0.0 (0.1) | 0.1 (0.1) | 0.521 | 0.811 |
| *Family_XIII_UCG-001* | 0.0 (0.1) | 0.0 (0.0) | 0.230 | 0.811 |
| *Flavonifractor* | 0.0 (0.0) | 0.0 (0.0) | 0.315 | 0.811 |
| *Fournierella* | 0.0 (0.0) | 0.0 (0.0) | 0.485 | 0.811 |
| *Frisingicoccus* | 0.0 (0.0) | 0.0 (0.0) | 0.485 | 0.811 |
| *Fructilactobacillus* | 0.0 (0.0) | 0.0 (0.0) | 0.640 | 0.811 |
| *Fusicatenibacter* | 1.7 (1.4) | 1.4 (1.3) | 0.500 | 0.811 |
| *Gastranaerophilales* | 0.0 (0.0) | 0.0 (0.0) | 0.381 | 0.811 |
| *GCA-900066575* | 0.0 (0.0) | 0.0 (0.0) | 0.381 | 0.811 |
| *Gordonibacter* | 0.0 (0.0) | 0.0 (0.0) | 0.827 | 0.922 |
| *Haemophilus* | 0.0 (0.1) | 0.0 (0.0) | 0.645 | 0.811 |
| *Holdemanella* | 1.4 (3.4) | 3.1 (3.3) | 0.178 | 0.811 |
| *Holdemania* | 0.0 (0.0) | 0.0 (0.0) | 0.304 | 0.811 |
| *Howardella* | 0.0 (0.0) | 0.0 (0.1) | 0.466 | 0.811 |
| *Incertae_Sedis* | 0.4 (0.5) | 0.2 (0.3) | 0.010 | 0.303 |
| *Intestinibacter* | 0.2 (0.5) | 0.5 (0.8) | 0.303 | 0.811 |
| *Intestinimonas* | 0.0 (0.0) | 0.0 (0.0) | 0.055 | 0.614 |
| *Izemoplasmatales* | 0.0 (0.0) | 0.0 (0.0) | 0.599 | 0.811 |
| *Lachnoclostridium* | 0.4 (0.5) | 0.7 (0.6) | 0.047 | 0.614 |
| *Lachnospira* | 0.2 (0.3) | 0.2 (0.3) | 0.471 | 0.811 |
| *Lachnospiraceae_FCS020_group* | 0.2 (0.3) | 0.2 (0.4) | 0.791 | 0.921 |
| *Lachnospiraceae_NC2004_group* | 0.1 (0.0) | 0.1 (0.0) | 0.312 | 0.811 |
| *Lachnospiraceae_ND3007_group* | 0.5 (0.3) | 0.4 (0.2) | 0.189 | 0.811 |
| *Lachnospiraceae_NK4A136_group* | 0.5 (0.7) | 0.8 (1.0) | 0.946 | 0.964 |
| *Lachnospiraceae_UCG-001* | 0.0 (0.2) | 0.0 (0.3) | 0.885 | 0.930 |
| *Lachnospiraceae_UCG-004* | 0.1 (0.0) | 0.1 (0.0) | 0.746 | 0.884 |
| *Lachnospiraceae_UCG-008* | 0.0 (0.0) | 0.0 (0.0) | 0.224 | 0.811 |
| *Lachnospiraceae_UCG-010* | 0.0 (0.1) | 0.0 (0.1) | 0.676 | 0.837 |
| *Lacticaseibacillus* | 0.0 (0.0) | 0.0 (0.0) | 0.305 | 0.811 |
| *Lactobacillus* | 0.0 (0.0) | 0.0 (0.0) | 0.485 | 0.811 |
| *Lactococcus* | 0.0 (0.0) | 0.0 (0.0) | 0.861 | 0.926 |
| *Latilactobacillus* | 0.0 (0.0) | 0.0 (0.0) | 0.381 | 0.811 |
| *Libanicoccus* | 0.0 (0.0) | 0.0 (0.0) | 0.616 | 0.811 |
| *Ligilactobacillus* | 0.0 (0.0) | 0.0 (0.0) | 0.197 | 0.811 |
| *Limosilactobacillus* | 0.0 (0.0) | 0.0 (0.0) | 0.635 | 0.811 |
| *Mailhella* | 0.0 (0.0) | 0.0 (0.0) | 0.640 | 0.811 |
| *Marvinbryantia* | 0.0 (0.0) | 0.0 (0.0) | 0.486 | 0.811 |
| *Megamonas* | 0.0 (0.0) | 0.0 (0.4) | 0.498 | 0.811 |
| *Megasphaera* | 0.0 (0.0) | 0.0 (0.1) | 0.131 | 0.811 |
| *Merdibacter* | 0.0 (0.0) | 0.0 (0.0) | 0.305 | 0.811 |
| *Methanobrevibacter* | 0.0 (0.3) | 0.0 (1.0) | 0.282 | 0.811 |
| *Methanosphaera* | 0.0 (0.0) | 0.0 (0.0) | 0.331 | 0.811 |
| *Mitsuokella* | 0.0 (0.0) | 0.0 (0.0) | 0.331 | 0.811 |
| *Mogibacterium* | 0.0 (0.0) | 0.0 (0.0) | 0.640 | 0.811 |
| *Monoglobus* | 0.5 (0.4) | 0.4 (0.5) | 0.477 | 0.811 |
| *Moryella* | 0.0 (0.0) | 0.0 (0.0) | 0.565 | 0.811 |
| *Muribaculaceae* | 0.0 (0.0) | 0.0 (0.0) | 0.966 | 0.972 |
| *Negativibacillus* | 0.0 (0.0) | 0.0 (0.0) | 0.420 | 0.811 |
| *NK4A214_group* | 0.2 (0.3) | 0.2 (0.2) | 0.386 | 0.811 |
| *Odoribacter* | 0.2 (0.2) | 0.1 (0.2) | 0.575 | 0.811 |
| *Olsenella* | 0.0 (0.1) | 0.0 (0.0) | 0.733 | 0.884 |
| *Oscillibacter* | 0.0 (0.1) | 0.0 (0.1) | 0.826 | 0.922 |
| *Oxalobacter* | 0.0 (0.0) | 0.0 (0.0) | 0.302 | 0.811 |
| *Parabacteroides* | 0.8 (0.6) | 0.8 (0.7) | 0.563 | 0.811 |
| *Paraprevotella* | 0.1 (0.5) | 0.4 (0.4) | 0.508 | 0.811 |
| *Parasutterella* | 0.0 (0.0) | 0.0 (0.0) | 0.939 | 0.962 |
| *Parvibacter* | 0.0 (0.0) | 0.0 (0.0) | 0.381 | 0.811 |
| *Peptococcus* | 0.0 (0.0) | 0.0 (0.0) | 0.953 | 0.964 |
| *Peptostreptococcaceae* | 0.0 (0.0) | 0.0 (0.0) | 0.640 | 0.811 |
| *Phascolarctobacterium* | 0.4 (1.3) | 0.5 (0.9) | 0.744 | 0.884 |
| *Phocea* | 0.0 (0.0) | 0.0 (0.0) | 0.640 | 0.811 |
| *Phoenicibacter* | 0.0 (0.0) | 0.0 (0.0) | 0.640 | 0.811 |
| *Porphyromonas* | 0.0 (0.0) | 0.0 (0.0) | 0.331 | 0.811 |
| *Prevotella* | 0.0 (0.0) | 0.0 (0.0) | 0.565 | 0.811 |
| *Prevotella_7* | 0.2 (1.1) | 0.1 (1.1) | 0.463 | 0.811 |
| *Prevotella_9* | 0.6 (5.4) | 2.8 (8.5) | 0.494 | 0.811 |
| *Prevotellaceae_Ga6A1_group* | 0.0 (0.0) | 0.0 (0.0) | 0.640 | 0.811 |
| *Prevotellaceae_NK3B31_group* | 0.0 (0.0) | 0.0 (0.0) | 0.197 | 0.811 |
| *RF39* | 0.1 (0.1) | 0.1 (0.1) | 0.783 | 0.921 |
| *Rikenellaceae_RC9_gut_group* | 0.0 (0.0) | 0.0 (0.0) | 0.597 | 0.811 |
| *Romboutsia* | 1.1 (1.7) | 1.3 (0.7) | 0.870 | 0.926 |
| *Roseburia* | 0.6 (1.7) | 1.0 (1.7) | 1.000 | 1.000 |
| *Rothia* | 0.0 (0.0) | 0.0 (0.0) | 0.640 | 0.811 |
| *Ruminococcus* | 1.3 (1.2) | 1.5 (1.3) | 0.863 | 0.926 |
| *Sanguibacteroides* | 0.0 (0.0) | 0.0 (0.0) | 0.640 | 0.811 |
| *Sarcina* | 0.0 (0.0) | 0.0 (0.0) | 0.635 | 0.811 |
| *Sellimonas* | 0.0 (0.0) | 0.0 (0.0) | 0.055 | 0.614 |
| *Senegalimassilia* | 0.0 (0.5) | 0.0 (0.3) | 0.367 | 0.811 |
| *Slackia* | 0.2 (0.3) | 0.4 (0.4) | 0.164 | 0.811 |
| *Solobacterium* | 0.0 (0.0) | 0.0 (0.0) | 0.331 | 0.811 |
| *Staphylococcus* | 0.0 (0.0) | 0.0 (0.0) | 0.055 | 0.614 |
| *Streptococcus* | 0.6 (1.0) | 0.3 (0.3) | 0.011 | 0.303 |
| *Subdoligranulum* | 3.0 (1.6) | 2.5 (0.9) | 0.241 | 0.811 |
| *Succinivibrio* | 0.0 (0.0) | 0.0 (0.0) | 0.640 | 0.811 |
| *Sutterella* | 0.2 (0.3) | 0.3 (0.4) | 0.188 | 0.811 |
| *Terrisporobacter* | 0.0 (0.0) | 0.2 (0.3) | 0.037 | 0.614 |
| *Turicibacter* | 0.0 (0.1) | 0.0 (0.1) | 0.883 | 0.930 |
| *Tyzzerella* | 0.0 (0.0) | 0.0 (0.0) | 0.501 | 0.811 |
| *UBA1819* | 0.0 (0.0) | 0.0 (0.0) | 0.169 | 0.811 |
| *UCG-002* | 0.6 (0.7) | 0.6 (0.7) | 0.939 | 0.962 |
| *UCG-003* | 0.0 (0.1) | 0.1 (0.2) | 0.129 | 0.811 |
| *UCG-005* | 0.1 (0.3) | 0.0 (0.3) | 0.737 | 0.884 |
| *UCG-008* | 0.0 (0.0) | 0.0 (0.0) | 0.302 | 0.811 |
| *UCG-009* | 0.0 (0.0) | 0.0 (0.0) | 0.485 | 0.811 |
| *UCG-010* | 0.1 (0.0) | 0.1 (0.1) | 0.361 | 0.811 |
| uncultured | 1.3 (0.6) | 1.1 (0.7) | 0.093 | 0.804 |
| *vadinBE97* | 0.0 (0.0) | 0.0 (0.0) | 0.055 | 0.614 |
| *Veillonella* | 0.0 (0.0) | 0.0 (0.0) | 0.381 | 0.811 |
| *Victivallis* | 0.0 (0.0) | 0.0 (0.0) | 0.006 | 0.238 |
| *Weissella* | 0.0 (0.0) | 0.0 (0.0) | 0.640 | 0.811 |

Between group differences are assessed by Mann-Whitney U and corrected for multiple comparisons using the Benjamini and Hochberg false discovery rate correction (*p*_adj_ value).

**Supplementary Table 12.** Biomarkers of gastrointestinal barrier integrity, and inflammation at baseline and post-heat tolerance assessment for heat tolerant (*n*=46) and heat intolerant individuals (*n*=12).

|  | **Heat tolerant** | | | **Heat intolerant** | | ***p* value; Effect size** | | |
| --- | --- | --- | --- | --- | --- | --- | --- | --- |
|  | | Baseline | Post-HTA | Baseline | Post-HTA ^a^ | Timepoint | Group | Interaction |
| I-FABP _Log_ (pg·mL^-1^) | | 6.38 ± 0.15 | 7.07 ± 0.15 | 5.90 ± 0.29 | 6.78 ± 0.29 | <0.001***; 0.52 | 0.213; 0.03 | 0.364; 0.01 |
| *I-FABP (pg·mL^-1^)* | | *421.85 (698.00)* | *1204.21 (1558.50)* | *326.49 (463.01)* | *903.04 (1368.42)^a^* | - | - | - |
| CLDN3 (ng·mL^-1^) | | 7.65 ± 0.29 | 6.69 ± 0.29 | 8.04 ± 0.57 | 6.80 ± 0.58 | <0.001***; 0.30 | 0.675; <0.01 | 0.539; 0.01 |
| LBP _Log_ (µg·mL^-1^) | | 1.04 ± 0.09 | 1.03 ± 0.09 | 1.01 ± 0.18 | 1.06 ± 0.18 | 0.225; 0.03 | 0.989; <0.01 | 0.110; 0.04 |
| *LBP (µg·mL^-1^)* | | *3.26 ± 1.49* | *3.23 ± 1.47* | *2.98 ± 1.35* | *3.24 ± 1.42* | - | - | - |
| sCD14 (µg·mL^-1^) | | 1.35 ± 0.05 | 1.34 ± 0.05 | 1.52 ± 0.09 | 1.65 ± 0.09 | 0.061; 0.06 | 0.014*; 0.10 | 0.058; 0.06 |
| IL-6 _Log_ (pg·mL^-1^) | | -0.52 ± 0.10 | 1.19 ± 0.10 | -0.60 ± 0.19 | 1.57 ± 0.20 | <0.001***; 0.82 | 0.377; 0.01 | 0.063; 0.06 |
| *IL-6 (pg·mL^-1^)* | | *0.57 (0.59)* | *3.23 (1.72)* | *0.42 (0.80)* | *4.93 (3.26)* | - | - | - |

Data are presented as estimated marginal means ± SE. Effect size is presented as partial eta squared (*η*^2^*_p_*). Where data has been log transformed, the raw data is presented as mean ± SD for parametric data, or median (IQR) for non-parametric data. I-FABP=intestinal fatty acid binding protein; CLDN-3=claudin 3; LBP=liposaccharide binding protein; sCD14=soluble Cluster of Differentiation 14; IL-6=interleukin 6 ^a^ *n*=11. **p=*<0.050; ***=*p*<0.001.

# Supplementary Figures

**
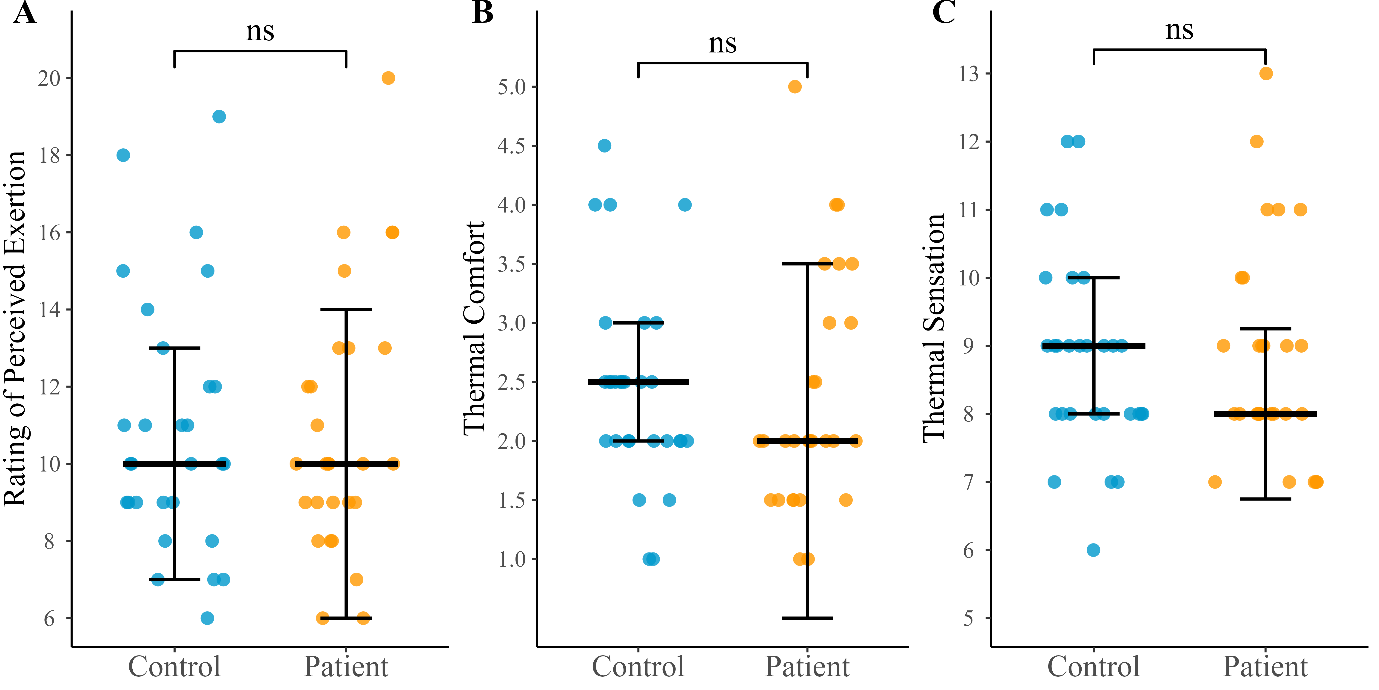
Supplementary Figure 1.** Terminal perceptual values during the heat tolerance assessment (HTA) for control participants within previous Exertional Heat Illness (EHI) history (Control: *n*=29) and recent EHI patients (Patient: *n*=28). Termination values are presented for Rating of Perceived Exertion (**A**), Thermal Comfort; (**B**), and Thermal Sensation (**C**). Large wide bar represents median (interquartile range). ns=not significant, *p*>0.05.


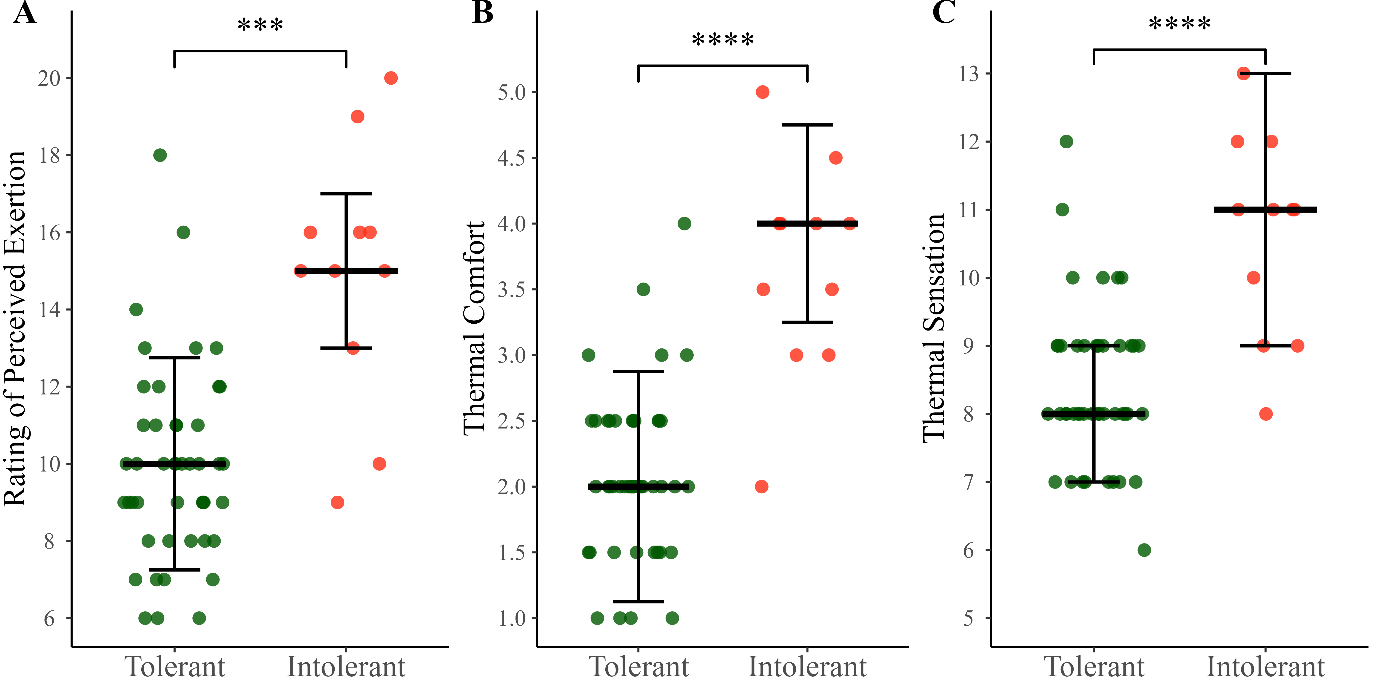


**Supplementary Figure 2.** Terminal perceptual responses during the heat tolerance assessment (HTA) for heat tolerant (*n*=46) and intolerant (*n*=11) individuals. Termination values are presented for Rating of Perceived Exertion (**A**), Thermal Comfort; (**B**), and Thermal Sensation (**C**). Large wide bar represents median (interquartile range). ns=not significant, *p*>0.05; ***=*p*<0.001; ****=*p*<0.0001.
